# Proteome Dynamics of the Specialist Oxalate Degrader *Oxalobacter formigenes*

**DOI:** 10.4172/jpb.1000384

**Published:** 2016

**Authors:** Melissa E Ellis, James A Mobley, Ross P Holmes, John Knight

**Affiliations:** Department of Urology, University of Alabama at Birmingham, Birmingham, AL, USA

**Keywords:** *Oxalobacter formigenes*, Calcium oxalate stone disease, Proteome dynamics

## Abstract

*Oxalobacter formigenes* is a unique intestinal organism that relies on oxalate degradation to meet most of its energy and carbon needs. A lack of colonization is a risk factor for calcium oxalate kidney stone disease. The release of the genome sequence of *O. formigenes* has provided an opportunity to increase our understanding of the biology of *O. formigenes*. This study used mass spectrometry based shotgun proteomics to examine changes in protein levels associated with the transition of growth from log to stationary phase. Of the 1867 unique protein coding genes in the genome of *O. formigenes* strain OxCC13, 1822 proteins were detected, which is at the lower end of the range of 1500–7500 proteins found in free-living bacteria. From the protein datasets presented here it is clear that *O. formigenes* contains a repertoire of metabolic pathways expected of an intestinal microbe that permit it to survive and adapt to new environments. Although further experimental testing is needed to confirm the physiological and regulatory processes that mediate adaptation with nutrient shifts, the *O. formigenes* protein datasets presented here can be used as a reference for studying proteome dynamics under different conditions and have significant potential for hypothesis development.

## Introduction

*Oxalobacter formigenes* is a Gram-negative, obligate anaerobic bacterium that commonly inhabits the human gut and degrades oxalate as its major energy and carbon source [1,2]. A review of colonization frequencies conducted worldwide indicated that 38–77% of a normal population is colonized with *O. formigenes* [3]. Recent evidence suggests a lack of colonization with *O. formigenes* may increase the risk for recurrent idiopathic calcium oxalate kidney stone disease [4,5]. Protection against calcium oxalate stone disease appears to be due to the oxalate degradation that occurs in the gut on low calcium diets [6] with a possible further contribution from intestinal oxalate secretion [7–9]. Despite the role this organism may play in reducing oxalate levels in the host and reducing the risk of calcium oxalate stone disease, there is scant information on how this organism colonizes the host and adapts to new environments. The release of the genome sequence of a Group 1 (OxCC13) and a Group 2 strain (HOxBLS) as part of the Human Microbiome Project has provided a genetic framework for investigating important biological properties of the organism [10]. In this study, we performed mass spectrometry (MS)-based shotgun proteomics of both log and stationary growth phase cultures of *O. formigenes*. These proteomic analyses of *O. formigenes* cultures provide insight into the physiological response associated with nutrient shifts and entry into stationary phase growth.

## Methods

### Culture conditions

Stages of *O. formigenes* growth in optimal laboratory broth culture conditions have been previously described [11]. Pure cultures of *O. formigenes*, strain OxCC13, were grown anaerobically at 37°C in 100 ml Schaedler broth (SBO, BD Biosciences) supplemented with 100 mM sodium oxalate and 10 mM sodium acetate. For generation of samples for proteomic analysis, *O. formigenes* cells were taken at OD_595_ 0.05 and 0.13 (n=4 each growth stage). These OD_595_ measurements correspond to mid-log and early stationary, and to 5.5 × 10^7^ and 1.4 × 10^8^ CFU/ml, respectively. Cells were washed three times with 0.9% saline prior to protein extraction.

#### Oxalate ion chromatography

Oxalate in culture media was quantified by ion chromatography (IC) using an AS22 2 mm column, as previously described [11].

### Proteomics experiments

**Figure 1:**
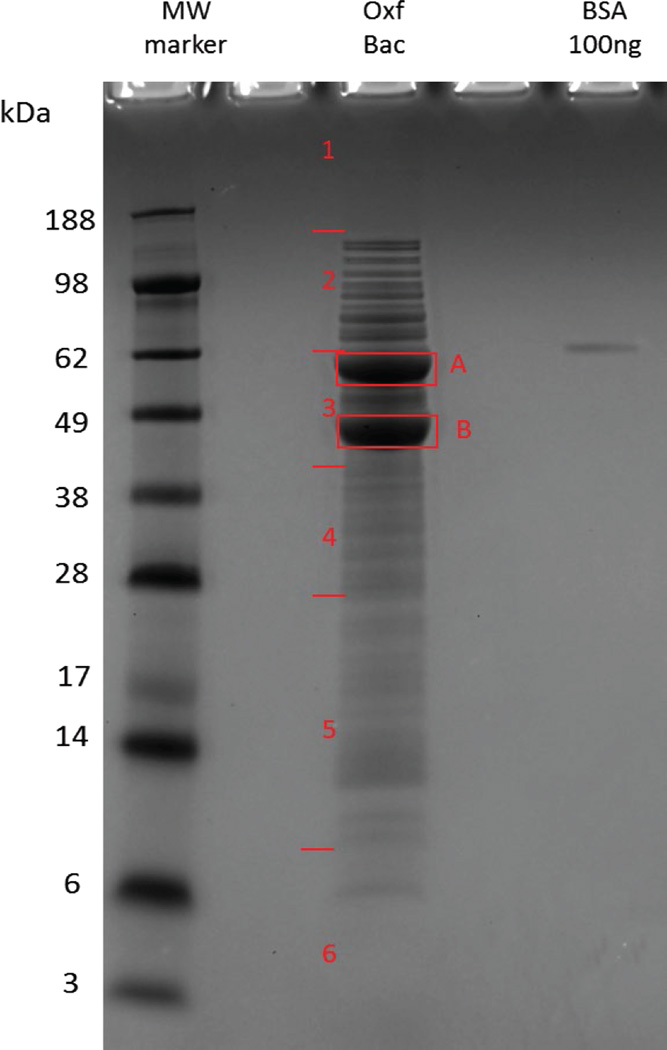
Representative gel of *O. formigenes* cell extract and areas excised for downstream MS analysis. *O. formigenes* cells (Oxf Bac); Bovine Serum Albumin (BSA).

Each cell pellet was lysed in B-per supplemented with lysozyme, Dnase I, and EDTA using the B-PER Kit (Pierce, Thermo Fisher Scientific) following manufacturers’ instructions. Protein concentrations of the cell lysates were determined with the BCA protein assay (Pierce, Thermo Fisher Scientific). Twenty micrograms (20 µg) of protein from each sample was diluted in LDS PAGE buffer (Invitrogen) followed by reducing, heat denaturing, and separation on a 10% SDS Bis-Tris gel (Invitrogen). The gel was stained overnight with Colloidal Blue (Invitrogen), and the two most abundant bands, A and B (Figure 1), were first carefully excised. Based on staining intensities, the rest of the gel lane was then cut into six nearly equal fractions from the top to bottom, and all eight of the resultant gel bands were then equilibrated in 100 mM ammonium bicarbonate (AmBc). Gel slices were reduced, carbidomethylated, dehydrated, and digested with Trypsin Gold (Promega) as per manufacturers’ instructions. Following digestion, peptides were extracted, volumes were reduced in a SpeedVac to near dryness, and re-suspended to 20 µL using 95% ddH_2_O/5% ACN/0.1% formic acid (FA) prior to analysis by 1D reverse phase LC-ESI-MS2 (as outlined below).

#### Mass spectrometry

Peptide digests were injected onto a Surveyor HPLC plus (Thermo Scientific) using a split flow configuration on the back end of a 100 micron I.D. × 13 cm pulled tip C-18 column (Jupiter C-18 300 Å, 5 micron, Phenomenex). This system runs in-line with a Thermo Orbitrap Velos Pro hybrid mass spectrometer, equipped with a nano-electrospray source (Thermo Scientific, San Jose CA), and all data were collected in CID mode. The HPLC was set up with two mobile phases that included solvent A (0.1% FA in ddH_2_O), and solvent B (0.1% FA in 85% ddH_2_O /15% ACN), programmed as follows; 15 min @ 0%B (2 µL/min, load), 65 min @ 0%–40% B (~0.5 nL/min, analyze), 20 min @ 0% B (2 µL/min, equilibrate). Following each parent ion scan (350–1200 m/z), fragmentation data was collected on the top most intense 15 ions. During data collection, the instrument was configured as follows: spray voltage 1.9 kV, capillary temperature 170°C, 1 microscan with a maximum inject time of 25 ms for all modes. The fragmentation scans were obtained at 60 K resolution with a minimum signal threshold of 2000 counts. The activation settings were set to charge state 3, isolation width 2.0 m/z, normalized collision energy 30.0, activation Q 0.250, and activation time 25 ms. For the dependent scans, charge state screening was enabled with 1+ ions excluded, and dynamic exclusion was enabled with the following settings: repeat count 2, repeat duration 15.0 s, exclusion list size 500, and exclusion duration 60.0 s.

#### MS Data conversion and searches

The XCalibur RAW files were collected in profile mode, centroided and converted to MzXML using ReAdW v. 3.5.1. The mgf files were then created using MzXML2Search (included in TPP v. 3.5) for all scans with a precursor mass between 350 Da and 1,200 Da. The data was searched using SEQUEST, which was set for three maximum missed cleavages, a precursor mass window of 20 ppm, trypsin digestion, variable modification C at 57.0293, and M at 15.9949. Searches were performed with a Human subset of the UniRef100 database.

#### Peptide filtering, grouping, and quantification

A list of peptide IDs were generated based on SEQUEST search results, which were filtered using Scaffold (Protein Sciences, Portland Oregon). Scaffold was applied in order to filter and group all of the matching peptides to generate and retain only high confidence IDs while also generating normalized spectral counts (N-SC’s) across all samples for the purpose of relative quantification. The filter cut-off values were set with peptide length (>5 AA’s), no peptides with a MH+1 charge state were included, peptide probabilities were calculated and set to >80% C.I., with the number of peptides per protein set at 2 or more, and protein probabilities were set to >99% C.I. and an FDR<1.0. Scaffold incorporates the two most common methods for statistical validation of large proteome datasets, the false discovery rate (FDR) and protein probability [12–14]. Relative quantification across experiments were then performed via spectral counting [15,16], and when relevant, spectral count abundances were then normalized between samples [17].

#### Statistical and systems analysis

For the proteomic data generated two separate non-parametric statistical analyses were performed between each pair wise comparison. These non-parametric analyses include 1) the calculation of weight values by significance analysis of microarray (SAM; cut off >|0.6|combined with 2) T-Test (single tail, unequal variance, cut off of *P*<0.05), which then were sorted according to the highest statistical relevance in each comparison. For SAM [18,19], whereby the weight value (W) is a statistically derived function that approaches significance as the distance between the means (μ1−μ2) for each group increases, and the SD (δ1−δ2) decreases using the formula, W= (μ1−μ2) / (δ1−δ2). For protein abundance ratios determined with N-SC’s, we set a 1.5-fold change as the threshold for significance, determined empirically by analyzing the inner-quartile data from the control experiment indicated above using ln-ln plots, where the Pierson’s correlation coefficient (R) was 0.98, and >99% of the normalized intensities fell between ± 1.5-fold. In each case, any two of the three tests (SAM, T-test, or fold change) had to pass.

## Results and Discussion

Culture medium oxalate concentration was determined at each phase of growth by ion chromatography. At log phase growth, media oxalate concentration was 54 ± 8 mM, indicating 54% of the oxalate in the culture medium had been degraded. At stationary phase, oxalate was not detected (<25 µM) indicating the cells were in a starved state.

Closer analysis of the annotated genome of *O. formigenes* strain OxCC13 revealed that 209 of the 2076 protein coding genes share the same gene annotation and amino acid sequence as other genes on different loci, indicating the genome of OxCC13 contains 1867 unique protein coding genes. Of these 1867 proteins, 663 (34%) are predicted or hypothetical proteins with no known function.

A proteomic dataset of *O. formigenes* OxCC13 was initially constructed from *O. formigenes* cells harvested from an early stationary culture (Supplemental Table S1). This dataset can be used as a reference for studying proteome dynamics under different conditions. This analysis identified 1822 proteins of the 1867 unique proteins of *O. formigenes* strain OxCC13. The proteomic approach was then used to identify proteome changes associated with the transition from log phase growth to stationary phase, offering a dynamic view of the different processes associated with entry into stationary phase.

Following gel electrophoresis, the two most abundant bands, A and B (Figure 1), were found to largely consist of oxalyl-CoA decarboxylase (569 amino acids) and formyl-coenzyme A transferase (429 amino acids), respectively, major proteins involved in oxalate catabolism [10]. The very high expression of these oxalate degrading proteins, highlighted in Figure 1, makes *O. formigenes* one of the most efficient oxalate degrading systems known. Of note, oxalyl-CoA decarboxylase (OFBG_01523), formyl-Co A transferase (OFBG_01036), and the oxalate (in):formate (out) antiporter OxlT (OFBG_01510), which is also central to oxalate metabolism in this organism, were all unchanged in log phase compared to stationary phase (Supplemental Table S2).

**Figure 2:**
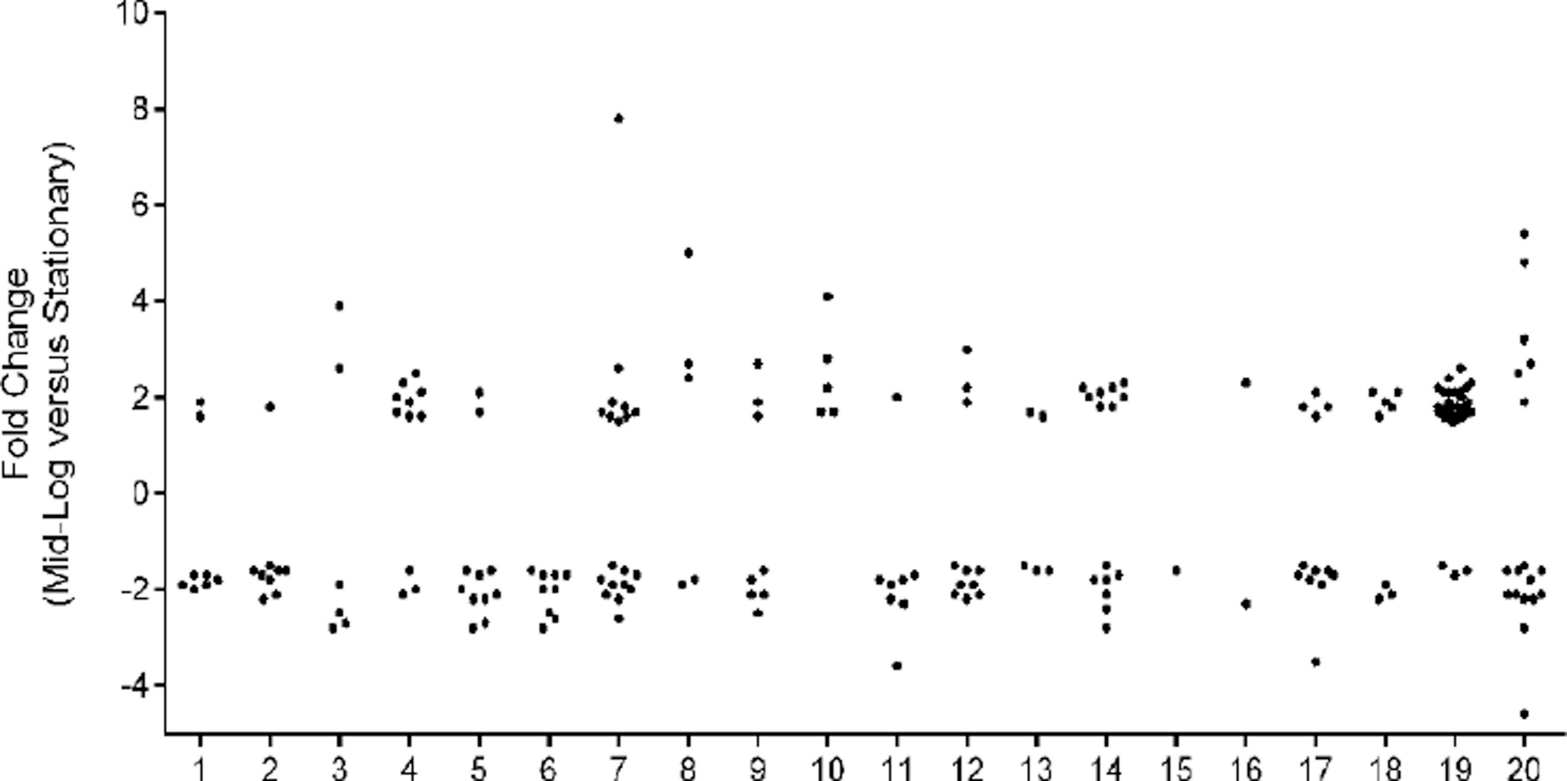
Fold change in protein levels of *O. formigenes* cells from log to stationary phase, as categorized by functional category. Functional categories: 1, Amino acid transport and metabolism; 2, Carbohydrate transport and metabolism; 3, Cell cycle control, cell division, chromosome partitioning; 4, Cell wall/membrane/envelope biogenesis; 5, Coenzyme transport and metabolism; 6, Defense mechanisms; 7, Energy production and conversion; 8, General function prediction only; 9, Inorganic ion transport and metabolism; 10, Intracellular trafficking, secretion, and vesicular transport; 11, Lipid transport and metabolism; 12, Nucleotide transport and metabolism; 13, Posttranslational modification, protein turnover, chaperones; 14, Replication, recombination and repair; 15, RNA processing and modification; 16, Secondary metabolites biosynthesis, transport and catabolism; 17, Signal transduction mechanisms; 18, Transcription; 19, Translation, ribosomal structure and biogenesis; 20, Unknown.

A total of 206 proteins with predicted function were found at significantly elevated or lowered levels when log was compared with stationary phase growth. Of these 206 proteins, 109 were detected at lower levels and 97 at higher levels, respectively, in cells from log phase versus stationary phase. The list of these 206 increased or decreased proteins with fold change (log versus stationary) and predicted functional category, membrane association, and molecular weight, are shown in Table S2 in the Supporting Information. Figure 2 gives the fold change in relative abundance from log to stationary of these 206 proteins, as categorized by predicted functional category.

### Log versus stationary phase

The majority of proteins that showed a higher relative abundance in log phase growth compared to stationary phase were involved in ribosomal structure and biogenesis, energy production, replication, and cell wall/membrane biogenesis (Figure 2 and Table S2). An ABC transporter like protein (OFBG_01114), an AAA-type ATPase (OFBG_00088), and a membrane bound pyrophosphate-energized proton pump (OFBG_02044) were more than 5-fold increase in log versus stationary cells. The major function of ABC import systems is to provide essential nutrients to bacteria, and AAA-type ATPases form a large protein family involved in a number of roles including protein proteolysis and disaggregation. The almost 8-fold increase in levels of a pyrophosphate-energized proton pump in log phase growth compared to stationary phase is in keeping with a proton motive force being the most important mechanism of energy production from oxalate catabolism in *O. formigenes* during maximal cell division [20]. Five proteins under the functional category, “Intracellular trafficking, secretion, and vesicular transport”, were elevated in log versus stationary, including a translocase SecD subunit protein (OFBG_01850), which is part of a protein export complex required to maintain a proton motive force, and three biopolymer transporter ExbB proteins (OFBG_02038, OFBG_02039 and OFBG_01048), which use a proton gradient across the inner bacterial membrane to transport large molecules across the outer bacterial membrane. These data together highlight the need of a proton motive force during maximal cell division.

As expected, ribosomal proteins were elevated in log phase relative to stationary (Figure 2). In *E. coli*, there are 21 small ribosomal proteins and 36 large ribosomal proteins. Many of the genes are encoded in an operon, and *O. formigenes* OxCC13 maintains this same structure. Proteomic analysis detected all these proteins, except S20/L26. The S20/L26 protein encoding gene is not annotated in the *O. formigenes* OxCC13 genome; however, is present in the HOxBLS genome (OFAG_01539). By comparing the location of OFAG_01539 with the OxCC13 genome, we identified OFBG_01579 (predicted protein) as the probable S20/L26 ribosomal protein encoding gene. Both genes are 273 nucleotides. DNA sequence alignment of HOxBLS gene OFAG_01539 with OxCC13 gene OFBG_01579 showed an 84% similarity, and the protein sequence was 94% similar suggesting that OxCC13 gene OFBG_01579 is in fact the 30S ribosomal protein S20 gene. Several, but not all, of the ribosomal proteins were significantly increased during log-phase growth.

### Stationary phase versus log phase growth

The protein found to increase the most in stationary relative to its level in log phase (4.6-fold increase, stationary versus log) was a peptidyl-prolyl-cis/trans-isomerase (PPIase) (OFBG_00644). The genome of *O. formigenes* contains 7 proteins with predicted PPIase activity. In addition to OFBG_00644, two other PPIases at loci OFBG_00237 and OFBG_01547 were also increased 2.2-fold and 1.6-fold, respectively, in stationary versus log phase. PPIases function as protein folding chaperones for proteins containing proline residues and have been found to be involved in a plethora of biological processes such as gene expression, signal transduction, protein degradation and protein secretion [21]. In bacteria, PPIases can be divided into soluble and membrane-bound subgroups. The PPIase at locus OFBG_00664 in *O. formigenes* is predicted to be membrane-bound, whereas the PPIases at loci OFBG_01547 and OFBG_00237 are predicted to be soluble proteins. OFBG-00644 and OFBG_00237 are periplasmic FKBP-type PPIases [22]. FKBP-type PPIases very often have a C-terminal PPIase and N-terminal domain usually involved in homodimerization, as is the case for FkpA of *Escherichia coli* [23]. OFBG_00644 seems to have a unique N-terminal domain. Further work to assess the function of the N-terminal domain of OFBG_00644 is of interest. OFBG_01547 shows homology with SurA, which in *E.coli* has been shown to be involved in the assembly of outer membrane porins [24], and in *Brucella abortus* is known to be secreted into the supernatant where it may play a role in virulence [25]. In prokaryotes, several PPIases are upregulated by stress factors, such as cold shock [26], due to accumulation of misfolded proteins under these conditions. Interestingly, deletion of all periplasmic PPIases of *Yersinia pseudotuberculosis*, despite showing no measurable phenotype under laboratory conditions, impaired colonization of mice [27]. These experiments and others indicate the contribution of PPIases to cellular physiology is likely to be very specific and dependent on growth conditions and the niche in which the cell resides. Examination of the role of PPIases to *O. formigenes* survival and colonization both *in vivo* and *in vitro* warrants further investigation.

There was a higher abundance of proteins predicted to be associated with defense mechanisms and stress resistance in stationary versus log phase. This is in keeping with studies with other Gram-negative bacteria, which have shown nutrient depletion in culture often leads to stress cross-protection; for example, the starvation response in *E. coli* also provides protection against osmotic stress [28]. Proteins associated with defense mechanisms and stress resistance that were increased in *O. formigenes* stationary cells included superoxide dismutase (OFBG_01322), an organic solvent tolerance protein (OFBG_01546), a toluene tolerance transporter (OFBG_01898), a ”resistance protein” (OFBG_01759) with good homology to osmotically induced protein C in *E. coli*, a HigA-like antidote protein (OFBG_00577), which is linked to persistence and dormancy upon exposure to stress, three universal stress proteins encoded by genes at loci OFBG_00781, OFBG_00128 and OFBG_01674, which have been shown to provide general “stress endurance” activity, an Abi family protein (OFBG_00600), which is involved in resisting bacteriophage infection, an esterase (OFBG_01018) with predicted beta-lactamase activity, and a transcriptional regulator of the TetR family (OFBG_00136), which are involved in the control of expression of multidrug resistance proteins. In Gram-negative organisms, enzymes belonging to the low molecular weight protein tyrosine phosphatase family are involved in regulation of important physiological functions including stress resistance [29]. The bacterial functions of low molecular weight protein tyrosine phosphatases remain largely unknown, although some hints are given by the organization of genes surrounding the protein tyrosine phosphatases on the chromosome [30]. The low molecular weight protein tyrosine phosphatase encoded by the gene at locus OFBG_00779 of *O. formigenes* was increased 3.5-fold in stationary relative to log phase. This gene is downstream of the antibiotic resistance gene encoding a 5-nitroimidazole antibiotic resistance protein (OFBG_00777) and may be involved in the increased expression of the 5-nitroimidazole antibiotic resistance protein in stationary phase. Proteomic analysis showed the 5-nitroimidazole antibiotic resistance protein to be increased 1.4-fold in stationary phase relative to log phase, although this was not significant (*P*=0.19). A number of proteins predicted to be involved with extrusion of heavy metals, including copper (OFBG_01218) and cobalt (OFBG_00614 and OFBG_00438), were also increased in expression in stationary phase relative to log phase. Three of the four predicted alcohol dehydrogenase proteins in *O. formigenes* were increased in stationary phase versus log phase, suggesting *O. formigenes* may be quite tolerant to alcohol exposure in stationary phase.

Transition to stationary phase from log growth resulted in increased expression of proteins associated with amino acid, carbohydrate, lipid, and nucleotide transport and metabolism, highlighting the ability of *O. formigenes* to utilize various carbon sources to survive when oxalate has been depleted. Proteins associated with carbohydrate transport and metabolism and increased in stationary versus log phase included a histidine-containing phosphocarrier transport protein (OFBG_01695), which transfers metabolic carbohydrates across the cell membrane, and various enzymes associated with glycolysis. A glycogen operon is present in the genome of *O. formigenes* (OFBG_01419 to 01424), and cells from stationary phase relative to log phase cells had higher levels of phosphoglucomutase (OFBG_01422), suggesting glycogen breakdown is important for prolonging survival of *O. formigenes* in the absence of oxalate. Proteins associated with metabolism of lipids included a thioesterase-like protein (OFBG_01426), a carboxylesterase-like protein (OFBG_01963), and malonate decarboxylase (OFBG_00828, gamma subunit, and OFBG_00827, delta subunit). The malonate decarboxylase gamma and delta subunits were increased 3.6 and 1.9-fold, respectively, in stationary versus log phase. Malonate decarboxylase catalyzes the degradation of malonate to acetate and CO_2_. Several strains of bacteria are able to utilize malonate as sole source of carbon and energy [31]. These data suggest acetate synthesis from malonate is an important mechanism by which *O. formigenes* survives in stationary phase.

Many of the proteins synthesized in *E. coli* cells in the early stages of starvation, as occurs in stationary phase, are proteases and peptidases [32]. The increased protein turnover facilitates *de novo* protein synthesis when exogenous carbon sources are low [33]. An ATP-dependent Lon protease (OFBG_00099) was increased in stationary phase cells relative to log, suggesting protein turnover may have increased in stationary phase cells.

A hallmark of stationary phase is the transformation to an enhanced barrier that includes extensive changes in the outer membrane, periplasm, and the inner membrane [34]. *O. formigenes* cells from stationary phase had increased levels of proteins involved with cell membrane biogenesis. These include a LolA-like protein (OFBG_01135), which is involved in outer membrane localization of lipoproteins [35], a predicted glycosyltransferase protein involved in lipopolysaccaride biosynthesis (OFBG_01135), and a membrane protein predicted to be involved in lipopolysaccharide assembly (OFBG_01972). Lysine biosynthesis was also increased in stationary relative to log (two genes in an operon involved in lysine biosynthesis, OFBG_01202 and OFBG_01207). Lysine is an important component of peptidoglycan and an increase in lysine synthesis may reflect the requirement for structural changes of the periplasm in stationary phase.

Biosynthesis of various cofactors and other important secondary metabolites was increased in stationary relative to log phase. These included proteins predicted to be involved in ubiquinone biosynthesis (OFBG_01584), molybdopterin synthesis (OFBG_01125 and OFBG_00896), inosine monophosphate biosynthesis (OFBG_01043), vitamin B12 biosynthesis (OFBG_01299 and OFBG_01907), and coenzyme A biosynthesis (OFBG_01787 and OFBG_02064). Vitamin B12 is an essential cofactor for several enzymes that catalyze a variety of transmethylation and rearrangement reactions [36]. Interestingly, an S-adenosyl-methionine-dependent methyltransferase (OFBG_01658) was also increased in stationary relative to log phase, suggesting the transfer of methyl groups to various biomolecules may play an important role in adaptive changes needed for the transition from log to stationary phase.

Proteins predicted to be associated with DNA repair were increased in stationary versus log phase, including a DNA exodeoxyribonuclease (OFBG_00812 beta subunit, and OFBG_00813, gamma subunit), a DNA end-binding protein Ku (OFBG_00801), a Holiday junction DNA helicase subunit RuvA (OFBG_01461), deoxyuridine 5’-triphosphate nucleotidohydrolase (OFBG_00045), a protein with sequence homology to MutT-like nucleoside triphosphate pyrophosphohydrolase (OFBG_01470), and two single strand DNA binding proteins (OFBG_01393 and OFBG_00293). Interestingly, a DNA mismatch repair protein MutL (OFBG_01061) was 1.8-fold lower in abundance in stationary phase compared to log phase. Studies with *E. coli* have shown that there is repression of genes in stationary phase associated with the methyl-mismatch repair (MMR) system, comprising genes *mutS, mutL* and *mutH* [37]. Repression of the MMR system induces the error-prone DNA pol IV and increases mutation rate. It has been hypothesized that because microorganisms in nature spend more of their life under stress conditions, stress-induced mutations could be an important way to generate genetic diversity, upon which natural selection will act to select the fittest mutant for a specific environmental condition.

In Gram-negative bacteria, entrance into stationary phase has been shown to be a very well- regulated process with sigma factors and many regulators involved [38]. Most of the regulatory mechanisms are complex and involve many regulatory links. The best example of this is the regulation of the alternate sigma factor RpoS, which governs entrance into stationary phase and stress resistance. Common transcriptional regulators whose expression levels are inversely related to growth rate in Gram-negative bacteria include leucine-responsive regulatory protein (Lrp) and integration host factor, commonly known as IHF. The genome of *O. formigenes* OxCC13 encodes proteins with significant amino acid sequence homology to *E. coli* RpoS (OFBG_01317), Lrp (OFBG_00685) and IHF (OFBG_00154). However, the proteins encoded by these genes were not increased significantly in stationary versus log phase. This may be because the genes in *O. formigenes* have different functions, or respond to later stages of stationary growth.

Other proteins predicted to be involved in “Nucleotide transport and metabolism” were increased in stationary versus log phase. Many of these proteins appear to be involved in the control of levels of nucleotide metabolic intermediates and signaling compounds (OFBG_00841, OFBG_00942, OFBG_00486, OFBG_00487, OFBG_00599, OFBG_01289). Proteins associated with degradation of stable RNA were also increased in stationary phase relative to log phase (OFBG_00227, OFBG_01664). The processing of nucleotides and degradation of stable RNA has been shown to occur in stationary phase and other forms of starvation in many bacterial species, and would appear to be of major importance to bacterial survival in such conditions, possibly by providing alternate sources of nitrogen and carbon [39,40].

Proteomic analysis detected 26 Sel1 repeat-containing proteins in *O. formigenes*, 8 of which were increased greater than 1.5-fold in stationary versus log phase, although only 5 significantly (*P*<0.05). In contrast, three Sel1 repeat-containing proteins were increased greater than 1.5-fold in log versus stationary phase (*P*<0.05). Proteins containing Sel1-like repeats mediate protein-protein interactions and are involved in a variety of biological processes including cell cycle regulation, transcriptional control and protein folding [41].

A number of proteins that play a role in signal transduction and transcriptional control changed in abundance in stationary phase. Two proteins with homology to histidine kinases (OFBG_01453 and OFBG_01663), which are known to be important to bacteria for sensing and responding to the outside and host environment [42], were increased in stationary relative to log phase. A transcriptional regulator (OFBG_01550) with a predicted LysR substrate binding domain was also increased 2-fold in stationary relative to log phase. The LysR family of transcriptional regulators represents the most abundant type of transcriptional regulator in the prokaryotic kingdom and regulates a diverse set of genes in stationary phase, including those involved in metabolism and quorum sensing [43].

## Concluding Remarks

The release of the genome sequence of *O. formigenes* has provided an opportunity for investigating important biological properties of *O. formigenes*. To date, no advanced omic studies in *O. formigenes* have been reported. As an initial approach to both enrich the annotation of the *O. formigenes* genome and identify proteins important for growth and survival, mass spectrometry based global shotgun proteomics was performed on *O. formigenes* OxCC13 cultures harvested from log phase and stationary phase. From the protein datasets presented here it is clear that *O. formigenes* contains numerous metabolic pathways that permits it to adapt to changing environments. Interestingly, the shift from abundant oxalate (log growth) to no oxalate (stationary phase) resulted in no change in the relative expression (log versus stationary) of the major proteins involved in oxalate catabolism. *O. formigenes* may retain high expression of these major oxalate catabolizing proteins in the absence of oxalate to ensure maximal growth when oxalate is reintroduced. This proteomic approach suggested superoxide dismutase increased in stationary relative to log phase suggesting *O. formigenes* has the ability to persist outside the anaerobic environment of the intestine. Furthermore, the increased abundance of three PPIases and the increase in utilization of malonate in stationary relative to log growth suggests these proteins are important for *O. formigenes* survival in stationary phase culture and possibly *in vivo*. Further experiments, including use of mutant strains, development of specific antibodies, and quantification of intracellular and extracellular metabolites, will be required to confirm these findings, and should lead to an improved understanding of how *O. formigenes* cells adapt and persist in various environments. Of interest was the recent filing of a patent by OxThera Pharmaceuticals that covers the invention of the isolation and administration of secretagogues derived from *O. formigenes* that may enhance oxalate secretion into the intestinal lumen (http://www.google.com/patents/WO2015002588A1?cl=en). The invention lists 19 possible secretagogues isolated from the cell free supernatant of a 400 liter culture. None of these potential secretagogues were increased or decreased in log phase relative to stationary phase. Future studies may identify proteins associated with host/*O. formigenes* interactions, including the mechanism by which *O. formigenes* enhances host oxalate secretion.

## Supplementary Material

Suppl Table 1

Suppl Table 2
