# Supplementary material for: Proteome Dynamics of the Specialist Oxalate Degrader Oxalobacter formigenes
Source: J Proteomics Bioinform. Author manuscript; Available in PMC 2016 Feb 24. (PMC4764995; doi:10.4172/jpb.1000384)
Supplement: Suppl Table 1 [file NIHMS758057-supplement-Suppl_Table_1.docx]

**Supplemental Table 1**: Identified proteins in *O. formigenes* strain OxCC13 with greater than or equal to 2 highly confident peptide ID’s per protein (>90% C.I.), with a final protein probability relating to >99% C.I. and an FDR<1%. Pure cultures were grown in rich broth medium (Schaedlers Broth) containing 100mM oxalate and were harvested at early stationary phase.

| **Protein Name** | **Accession #** | **Calculated MW** | **Unique**  **Peptides** |
| --- | --- | --- | --- |
| oxalyl-CoA decarboxylase | 1523 | 61 kDa | 59 |
| TCP-1/cpn60 chaperonin family protein | 1979 | 58 kDa | 64 |
| formyl-coenzyme A transferase | 1036 | 47 kDa | 48 |
| DNA-directed RNA polymerase subunit beta | 1991 | 157 kDa | 137 |
| ATP-dependent Clp protease ATP-binding subunit ClpB | 1106 | 97 kDa | 93 |
| glutamate dehydrogenase | 1435 | 49 kDa | 38 |
| elongation factor Tu | 2070 | 39 kDa | 41 |
| DNA-directed RNA polymerase subunit beta | 1990 | 153 kDa | 87 |
| small subunit ribosomal protein S1 | 1626 | 63 kDa | 55 |
| elongation factor G | 1994 | 78 kDa | 62 |
| hsp70-like protein | 2052 | 70 kDa | 65 |
| alkyl hydroperoxide reductase/Thiol specific antioxidant/Mal allergen | 67 | 21 kDa | 18 |
| translation initiation factor IF-2 | 1225 | 107 kDa | 94 |
| F0F1-type ATP synthase | 1823 | 50 kDa | 34 |
| formate-tetrahydrofolate ligase | 1536 | 60 kDa | 43 |
| S-adenosylhomocysteine hydrolase | 1728 | 52 kDa | 29 |
| tartronate-semialdehyde synthase | 1737 | 63 kDa | 41 |
| pyruvate carboxylase | 1739 | 138 kDa | 74 |
| 2-dehydropantoate 2-reductase | 2068 | 36 kDa | 23 |
| F0F1-type ATP synthase | 1825 | 56 kDa | 32 |
| methionine synthase | 1031 | 86 kDa | 56 |
| malate synthase G | 1465 | 81 kDa | 52 |
| acetate-CoA ligase | 291 | 73 kDa | 39 |
| carbamoyl phosphate synthase | 1167 | 119 kDa | 62 |
| malic enzyme | 1808 | 47 kDa | 31 |
| aspartyl-tRNA synthetase | 1953 | 68 kDa | 55 |
| large subunit ribosomal protein L1 | 1987 | 24 kDa | 19 |
| tartronic semialdehyde reductase | 1735 | 30 kDa | 22 |
| serine-pyruvate transaminase | 2069 | 45 kDa | 29 |
| elongation factor Ts | 267 | 32 kDa | 31 |
| small subunit ribosomal protein S5 | 2013 | 18 kDa | 23 |
| large subunit ribosomal protein L3 | 1996 | 23 kDa | 24 |
| tyrosine phenol-lyase | 1741 | 55 kDa | 42 |
| succinyl-CoA synthetase subunit beta | 1562 | 42 kDa | 39 |
| ribonuclease E protein | 889 | 108 kDa | 57 |
| translocase subunit secA | 1642 | 104 kDa | 65 |
| heat shock protein 90 | 1203 | 73 kDa | 55 |
| inosine-5'-monophosphate dehydrogenase | 1121 | 52 kDa | 38 |
| phosphoribosylformylglycinamidine synthase | 1185 | 144 kDa | 58 |
| peptidyl-prolyl cis/trans isomerase | 261 | 50 kDa | 37 |
| phosphoenolpyruvate carboxylase | 163 | 109 kDa | 53 |
| large subunit ribosomal protein L5 | 2008 | 20 kDa | 27 |
| methionine adenosyltransferase 1 | 71 | 42 kDa | 26 |
| pyridine nucleotide-disulphide oxidoreductase class-II | 66 | 56 kDa | 27 |
| alanyl-tRNA synthetase | 1184 | 96 kDa | 53 |
| ribonucleoside-triphosphate reductase | 1746 | 78 kDa | 34 |
| RNA polymerase primary sigma factor | 1317 | 91 kDa | 47 |
| polyribonucleotide nucleotidyltransferase | 108 | 77 kDa | 40 |
| formyl-CoA transferase | 2073 | 17 kDa | 17 |
| cyclopropane-fatty-acyl-phospholipid synthase | 1193 | 47 kDa | 37 |
| aconitate hydratase 1 | 167 | 99 kDa | 48 |
| modification methylase bstVI | 1842 | 60 kDa | 32 |
| type II citrate synthase | 450 | 49 kDa | 35 |
| dihydroxy-acid dehydratase 1 | 2036 | 66 kDa | 35 |
| isoleucyl-tRNA synthetase | 48 | 110 kDa | 59 |
| 2-isopropylmalate synthase | 104 | 55 kDa | 33 |
| DNA helicase | 476 | 226 kDa | 58 |
| small subunit ribosomal protein S3 | 2002 | 31 kDa | 32 |
| large subunit ribosomal protein L2 | 1999 | 30 kDa | 24 |
| DNA topoisomerase III | 1599 | 98 kDa | 57 |
| phospho-2-dehydro-3-deoxyheptonate aldolase | 1675 | 39 kDa | 27 |
| 3-isopropylmalate dehydratase large subunit | 1356 | 50 kDa | 25 |
| small subunit ribosomal protein S4 | 2020 | 24 kDa | 25 |
| thiamine biosynthesis protein thiC | 1637 | 71 kDa | 44 |
| 50S ribosomal subunit protein L6/L9E | 2011 | 19 kDa | 19 |
| argininosuccinate synthase | 1668 | 46 kDa | 24 |
| D-xylulose 5-phosphate/D-fructose 6-phosphate phosphoketolase | 1713 | 90 kDa | 46 |
| leucyl-tRNA synthetase | 1973 | 100 kDa | 48 |
| O-acetylhomoserine/O-acetylserine sulfhydrylase | 61 | 46 kDa | 21 |
| ketol-acid reductoisomerase | 21 | 37 kDa | 28 |
| adenylosuccinate synthetase | 200 | 47 kDa | 29 |
| acetolactate synthase large subunit | 23 | 63 kDa | 33 |
| 5,10-methylenetetrahydrofolate reductase | 1726 | 33 kDa | 20 |
| small subunit ribosomal protein S7 | 1993 | 18 kDa | 18 |
| multifunctional protein CAD | 1168 | 41 kDa | 20 |
| NADH peroxidase | 1250 | 61 kDa | 34 |
| DNA gyrase subunit B | 1797 | 93 kDa | 42 |
| translation elongation factor P | 1091 | 21 kDa | 13 |
| helicase | 1866 | 128 kDa | 48 |
| cysteine desulfurase | 36 | 47 kDa | 28 |
| DNA-binding ATP-dependent protease La | 264 | 89 kDa | 46 |
| transketolase | 1926 | 73 kDa | 43 |
| threonyl-tRNA synthetase | 148 | 72 kDa | 47 |
| anaerobic dehydrogenase | 54 | 80 kDa | 38 |
| DNA gyrase subunit A | 75 | 98 kDa | 50 |
| transcription termination factor Rho | 131 | 47 kDa | 33 |
| outer membrane protein | 275 | 20 kDa | 19 |
| phosphoglycerate kinase | 1200 | 43 kDa | 32 |
| glutamate synthase subunit large | 1384 | 172 kDa | 46 |
| 30S ribosomal subunit protein S2 | 266 | 27 kDa | 22 |
| aminopeptidase N | 1238 | 101 kDa | 39 |
| valyl-tRNA synthetase | 1638 | 106 kDa | 44 |
| B12-dependent methionine synthase | 1780 | 139 kDa | 47 |
| large subunit ribosomal protein L24 | 2007 | 11 kDa | 15 |
| peptidyl-prolyl cis-trans isomerase | 1220 | 72 kDa | 41 |
| glutaminyl-tRNA synthetase | 1500 | 67 kDa | 40 |
| aldehyde dehydrogenase | 55 | 52 kDa | 30 |
| lysyl-tRNA synthetase | 248 | 58 kDa | 37 |
| diaminopimelate dehydrogenase | 1871 | 33 kDa | 22 |
| small subunit ribosomal protein S6 | 1096 | 14 kDa | 16 |
| biotin carboxylase | 1575 | 50 kDa | 21 |
| acetyl-CoA hydrolase | 1879 | 55 kDa | 24 |
| DNA polymerase III alpha subunit | 218 | 129 kDa | 48 |
| large subunit ribosomal protein L16 | 2003 | 16 kDa | 12 |
| phosphoenolpyruvate synthase | 283 | 89 kDa | 41 |
| homoserine dehydrogenase | 1101 | 47 kDa | 27 |
| histidinol dehydrogenase | 1892 | 47 kDa | 26 |
| ribosomal protein S10 | 1995 | 12 kDa | 11 |
| CTP synthetase | 929 | 61 kDa | 26 |
| isocitrate dehydrogenase | 1848 | 46 kDa | 25 |
| molybdopterin oxidoreductase | 52 | 75 kDa | 39 |
| ATP-dependent protease ATP-binding subunit | 263 | 47 kDa | 25 |
| outer membrane porin protein | 1296 | 40 kDa | 18 |
| large subunit ribosomal protein L7/L12 | 1989 | 13 kDa | 15 |
| molecular chaperone DnaJ | 2053 | 41 kDa | 31 |
| glycyl-tRNA synthetase subunit beta | 1241 | 77 kDa | 38 |
| bifunctional purine biosynthesis protein purH | 1459 | 56 kDa | 36 |
| ATP-dependent protease ATP-binding subunit | 1929 | 50 kDa | 28 |
| MadA protein | 420 | 293 kDa | 38 |
| ATP-dependent helicase | 94 | 149 kDa | 54 |
| two-component hybrid sensor kinase/response regulator | 587 | 121 kDa | 40 |
| ribosomal protein L14 | 2006 | 13 kDa | 14 |
| N-methylhydantoinase | 1041 | 60 kDa | 26 |
| phosphorylase | 1419 | 93 kDa | 44 |
| serine hydroxymethyltransferase | 1755 | 46 kDa | 18 |
| adenylosuccinate lyase | 1539 | 53 kDa | 29 |
| 30S ribosomal subunit protein S13 | 2018 | 14 kDa | 21 |
| leucyl aminopeptidase | 90 | 54 kDa | 30 |
| enolase | 927 | 46 kDa | 24 |
| threonine synthase | 1127 | 55 kDa | 29 |
| fructose-1,6-bisphosphate aldolase | 1199 | 39 kDa | 17 |
| large subunit ribosomal protein L4 | 1997 | 23 kDa | 16 |
| 2-oxoglutarate dehydrogenase E2 component | 454 | 50 kDa | 27 |
| conserved hypothetical protein | 475 | 50 kDa | 29 |
| sun family protein | 1042 | 47 kDa | 30 |
| argininosuccinate lyase | 2037 | 52 kDa | 31 |
| transcription-repair coupling factor | 1137 | 130 kDa | 46 |
| predicted protein | 1824 | 32 kDa | 26 |
| 50S ribosomal subunit protein L11 | 1986 | 15 kDa | 12 |
| small subunit ribosomal protein S9 | 40 | 14 kDa | 12 |
| aspartokinase | 127 | 45 kDa | 23 |
| transcription elongation protein | 1226 | 58 kDa | 31 |
| 3-isopropylmalate dehydrogenase | 1354 | 41 kDa | 23 |
| signal recognition particle | 1535 | 49 kDa | 32 |
| glyceraldehyde-3-phosphate dehydrogenase | 1927 | 36 kDa | 20 |
| DNA polymerase III subunit beta | 1796 | 41 kDa | 24 |
| arginyl-tRNA synthetase | 1845 | 63 kDa | 34 |
| nucleoside diphosphate kinase | 187 | 15 kDa | 14 |
| UvrABC system protein A | 1394 | 105 kDa | 45 |
| ATP-dependent Clp protease ATP-binding subunit ClpA | 1606 | 85 kDa | 31 |
| ABC transporter | 1903 | 62 kDa | 30 |
| homospermidine synthase | 1057 | 52 kDa | 29 |
| outer membrane porin protein | 1751 | 26 kDa | 10 |
| 30S ribosomal subunit protein S14 | 2009 | 12 kDa | 12 |
| seryl-tRNA synthetase | 1436 | 48 kDa | 26 |
| formyl-coenzyme A transferase | 2072 | 5 kDa | 3 |
| ferritin and Dps | 247 | 22 kDa | 11 |
| aspartate aminotransferase | 1102 | 45 kDa | 23 |
| prolyl-tRNA synthetase | 1533 | 66 kDa | 37 |
| DNA-binding ATP-dependent protease La | 99 | 89 kDa | 29 |
| anthranilate synthase component I | 1593 | 55 kDa | 27 |
| hydroxypyruvate isomerase | 1736 | 29 kDa | 17 |
| glutamine synthetase | 239 | 53 kDa | 22 |
| peptidase S41A | 1686 | 52 kDa | 26 |
| small subunit ribosomal protein S12 | 1992 | 14 kDa | 15 |
| DNA-directed RNA polymerase subunit alpha | 2021 | 36 kDa | 21 |
| phenylalanyl-tRNA synthetase subunit beta | 153 | 90 kDa | 29 |
| rod shape-determining protein mreB | 1306 | 37 kDa | 22 |
| preprotein translocase YidC subunit | 1791 | 63 kDa | 32 |
| fumarylacetoacetate hydrolase | 1847 | 28 kDa | 20 |
| threonine dehydratase | 1945 | 56 kDa | 26 |
| glutamate-5-semialdehyde dehydrogenase | 1970 | 46 kDa | 25 |
| acriflavine resistance protein A | 135 | 45 kDa | 21 |
| GMP synthase | 1120 | 59 kDa | 28 |
| histidine tRNA synthetase | 191 | 51 kDa | 25 |
| chromosome segregation protein | 202 | 134 kDa | 29 |
| glycosyltransferase 36 | 254 | 333 kDa | 41 |
| amidophosphoribosyltransferase | 1339 | 56 kDa | 25 |
| GGDEF domain-containing protein | 1481 | 227 kDa | 43 |
| tryptophan synthase subunit beta | 1690 | 44 kDa | 26 |
| ribosomal large subunit pseudouridine synthase B | 256 | 56 kDa | 43 |
| methionyl-tRNA synthetase | 1265 | 78 kDa | 31 |
| glutamyl-tRNA | 1308 | 53 kDa | 28 |
| ribosome recycling factor | 269 | 21 kDa | 21 |
| acetyl-CoA carboxylase subunit beta | 1343 | 32 kDa | 26 |
| chaperone SurA | 1547 | 52 kDa | 27 |
| large subunit ribosomal protein L17 | 2022 | 15 kDa | 13 |
| ATP-dependent RNA helicase | 217 | 52 kDa | 24 |
| AAA ATPase | 1484 | 62 kDa | 25 |
| tex transcriptional accessory protein | 1867 | 87 kDa | 34 |
| thiamine biosynthesis protein | 1635 | 28 kDa | 19 |
| glutamine-synthetase adenylyltransferase | 1679 | 105 kDa | 29 |
| preprotein translocase SecD subunit | 1850 | 68 kDa | 23 |
| cell division protease | 1163 | 69 kDa | 31 |
| 50S ribosomal protein L31 type B | 1188 | 10 kDa | 11 |
| acetolactate synthase isozyme III small subunit | 22 | 18 kDa | 10 |
| 23S rRNA 5-methyluridine methyltransferase | 159 | 52 kDa | 23 |
| phosphoribosylglycinamide synthetase | 232 | 46 kDa | 24 |
| DNA-damage-inducible protein D | 340 | 28 kDa | 18 |
| aromatic-amino-acid transaminase | 1085 | 45 kDa | 20 |
| GTP-binding elongation factor family protein | 1222 | 68 kDa | 24 |
| DnaK suppressor protein | 1300 | 19 kDa | 12 |
| hydroxypyruvate reductase | 1734 | 45 kDa | 21 |
| Zn-dependent oligopeptidase | 1957 | 77 kDa | 27 |
| ribosomal RNA large subunit methyltransferase N | 188 | 43 kDa | 24 |
| tryptophanyl-tRNA synthetase | 1169 | 46 kDa | 30 |
| FtsZ cell division protein | 1645 | 42 kDa | 17 |
| alpha-ketoglutarate decarboxylase | 453 | 107 kDa | 34 |
| cysteine synthase A | 527 | 32 kDa | 15 |
| oxalate:formate antiport protein | 1510 | 45 kDa | 7 |
| fumarase A | 290 | 55 kDa | 31 |
| glutamyl-tRNA amidotransferase subunit B | 1309 | 54 kDa | 22 |
| pyruvate kinase | 1733 | 51 kDa | 20 |
| ribose-phosphate pyrophosphokinase | 1935 | 35 kDa | 17 |
| small subunit ribosomal protein S17 | 2005 | 10 kDa | 10 |
| large subunit ribosomal protein L18 | 2012 | 13 kDa | 10 |
| recombinase A | 2033 | 38 kDa | 18 |
| GTP pyrophosphokinase | 1468 | 84 kDa | 33 |
| 50S ribosomal subunit protein L25 | 1936 | 22 kDa | 12 |
| exoribonuclease RNAse R | 115 | 89 kDa | 34 |
| ABC transporter ATP-binding protein | 1114 | 60 kDa | 31 |
| major facilitator transporter | 1262 | 59 kDa | 9 |
| large subunit ribosomal protein L19 | 1471 | 15 kDa | 13 |
| DNA mismatch repair protein mutS | 17 | 99 kDa | 34 |
| biotin synthase | 1621 | 39 kDa | 24 |
| F0F1 ATP synthase subunit B | 1827 | 17 kDa | 13 |
| phenylalanyl-tRNA synthetase subunit alpha | 152 | 38 kDa | 16 |
| predicted protein | 1857 | 11 kDa | 10 |
| PpiC-type peptidyl-prolyl cis-trans isomerase | 1955 | 29 kDa | 20 |
| large subunit ribosomal protein L22 | 2001 | 12 kDa | 12 |
| acetoin utilization protein | 1040 | 50 kDa | 22 |
| LSU ribosomal protein L9P | 1099 | 16 kDa | 13 |
| adenylate kinase | 2043 | 24 kDa | 14 |
| molecular chaperone GrpE | 2051 | 21 kDa | 17 |
| dehydrogenase | 1123 | 35 kDa | 20 |
| topoisomerase IV subunit A | 1229 | 86 kDa | 37 |
| DNA polymerase I | 1816 | 103 kDa | 28 |
| SmpA/OmlA family protein | 1975 | 46 kDa | 24 |
| large subunit ribosomal protein L15 | 2015 | 15 kDa | 10 |
| gamma-glutamyltranspeptidase | 1438 | 64 kDa | 23 |
| succinyl-CoA synthetase subunit alpha | 1561 | 31 kDa | 17 |
| tRNA modification GTPase mnmE | 1722 | 50 kDa | 18 |
| bifunctional 3-dehydroquinate synthase/shikimate kinase | 2029 | 61 kDa | 30 |
| cysteinyl-tRNA synthetase | 123 | 52 kDa | 28 |
| CoA-binding protein | 979 | 15 kDa | 15 |
| predicted protein | 1980 | 10 kDa | 11 |
| pantothenate synthetase | 1268 | 32 kDa | 16 |
| dihydrodipicolinate synthase | 1382 | 31 kDa | 15 |
| H+-transporting two-sector ATPase | 1822 | 15 kDa | 6 |
| small subunit ribosomal protein S11 | 2019 | 14 kDa | 8 |
| conserved hypothetical protein | 44 | 40 kDa | 13 |
| glutamate synthase oxidoreductase | 1383 | 53 kDa | 21 |
| orotate phosphoribosyltransferase | 1688 | 26 kDa | 13 |
| glucosamine-fructose-6-phosphate aminotransferase | 1750 | 67 kDa | 28 |
| DNA ligase | 204 | 76 kDa | 24 |
| predicted protein | 421 | 223 kDa | 29 |
| Sel1 repeat-containing protein | 544 | 139 kDa | 31 |
| 5-amino-6- | 1754 | 40 kDa | 26 |
| acriflavin resistance transmembrane protein | 134 | 118 kDa | 25 |
| uridylate kinase | 268 | 25 kDa | 11 |
| histidinol-phosphate aminotransferase | 78 | 40 kDa | 15 |
| S-malonyltransferase | 178 | 32 kDa | 14 |
| Sel1 repeat-containing protein | 545 | 132 kDa | 33 |
| ATP-binding domain-containing protein | 1264 | 40 kDa | 13 |
| tyrosyl-tRNA synthetase | 1448 | 46 kDa | 21 |
| ferritin and Dps | 1865 | 20 kDa | 13 |
| UDP-N-acetylglucosamine 1-carboxyvinyltransferase | 1894 | 45 kDa | 14 |
| ribonucleotide-diphosphate reductase subunit alpha | 18 | 85 kDa | 25 |
| NifU-like protein | 35 | 14 kDa | 13 |
| 3-oxoacyl-[acyl-carrier-protein] synthase II | 181 | 43 kDa | 17 |
| ATP phosphoribosyltransferase regulatory subunit | 199 | 43 kDa | 22 |
| dihydrolipoamide dehydrogenase | 455 | 51 kDa | 19 |
| microcin B17 maturation protein | 1676 | 52 kDa | 20 |
| ADP-L-glycero-D-mannoheptose-6-epimerase | 574 | 38 kDa | 17 |
| aspartate-semialdehyde dehydrogenase | 1353 | 41 kDa | 15 |
| ATP phosphoribosyltransferase catalytic subunit | 1893 | 25 kDa | 11 |
| 3-oxoacyl-[acyl-carrier-protein] synthase III | 177 | 35 kDa | 16 |
| topoisomerase IV subunit B | 1228 | 73 kDa | 23 |
| LSU ribosomal protein L13P | 41 | 16 kDa | 14 |
| predicted protein | 189 | 35 kDa | 17 |
| predicted protein | 649 | 54 kDa | 15 |
| ribonucleotide reductase large subunit | 1180 | 106 kDa | 28 |
| small subunit ribosomal protein S21 | 1320 | 9 kDa | 9 |
| phosphoglucomutase | 1422 | 59 kDa | 24 |
| GTPase ObgE | 1529 | 40 kDa | 20 |
| UDP-N-acetylmuramoylalanyl-D-glutamate-2,6-diaminopimelate ligase | 1655 | 55 kDa | 22 |
| acetyltransferase | 820 | 17 kDa | 11 |
| outer membrane cobalamin receptor | 1272 | 70 kDa | 21 |
| ATP-binding ABC transporter | 1525 | 72 kDa | 21 |
| acetylornithine aminotransferase | 1721 | 43 kDa | 15 |
| TPR repeat-containing protein | 81 | 68 kDa | 22 |
| ATPase | 88 | 62 kDa | 18 |
| transaldolase B | 138 | 35 kDa | 17 |
| cation-transporting P-ATPase | 749 | 99 kDa | 24 |
| dihydroorotase | 1291 | 46 kDa | 17 |
| tRNA uridine 5-carboxymethylaminomethyl modification enzyme mnmG | 1807 | 71 kDa | 22 |
| N-acetyl-gamma-glutamyl-phosphate reductase | 39 | 38 kDa | 12 |
| chorismate mutase | 77 | 39 kDa | 15 |
| conserved hypothetical protein | 233 | 27 kDa | 14 |
| secretion protein HlyD | 1146 | 34 kDa | 21 |
| translocation protein TolB | 1280 | 46 kDa | 24 |
| glutamate N-acetyltransferase | 1641 | 44 kDa | 15 |
| FtsA cell division protein | 1646 | 44 kDa | 16 |
| cell division protein | 203 | 41 kDa | 12 |
| arginine decarboxylase | 388 | 86 kDa | 17 |
| flavoprotein NADH-dependent oxidoreductase | 1314 | 40 kDa | 15 |
| glmU protein | 1714 | 48 kDa | 21 |
| 2-nitropropane dioxygenase | 129 | 46 kDa | 22 |
| aldo/keto reductase | 1497 | 40 kDa | 17 |
| methionyl-tRNA formyltransferase | 1602 | 34 kDa | 13 |
| pyruvate dehydrogenase E2 component | 1611 | 49 kDa | 19 |
| pyruvate dehydrogenase subunit E1 | 1612 | 101 kDa | 28 |
| predicted protein | 1701 | 15 kDa | 17 |
| phosphopantothenoylcysteine decarboxylase | 46 | 43 kDa | 16 |
| 30S ribosomal subunit protein S15 | 107 | 10 kDa | 11 |
| acetyl-coenzyme A carboxylase carboxyl transferase subunit alpha | 125 | 36 kDa | 16 |
| 3-oxoacyl-[acyl-carrier protein] reductase | 179 | 26 kDa | 9 |
| phosphomethylpyrimidine kinase | 945 | 29 kDa | 11 |
| hydroxyacylglutathione hydrolase | 1211 | 30 kDa | 13 |
| molecular chaperone HscA | 32 | 68 kDa | 28 |
| conserved hypothetical protein | 367 | 29 kDa | 15 |
| ATP-dependent RNA helicase | 1257 | 52 kDa | 20 |
| conserved hypothetical protein | 1439 | 21 kDa | 11 |
| phospholipid-binding protein | 1455 | 23 kDa | 10 |
| saicar synthetase | 856 | 26 kDa | 13 |
| fructose-bisphosphatase | 1239 | 38 kDa | 20 |
| DNA polymerase III subunit gamma | 1583 | 66 kDa | 22 |
| phosphoenolpyruvate-protein phosphotransferase | 1694 | 65 kDa | 19 |
| major facilitator transporter | 1731 | 46 kDa | 9 |
| ribosomal protein L27 | 1528 | 9 kDa | 5 |
| FAD-dependent pyridine nucleotide-disulphide oxidoreductase | 259 | 39 kDa | 17 |
| helicase | 402 | 112 kDa | 22 |
| diguanylate cyclase/phosphodiesterase with PAS/PAC and GAF sensor | 755 | 76 kDa | 24 |
| FtsK/SpoIIIE family DNA segregation ATPase | 1134 | 86 kDa | 22 |
| chromosomal replication initiator protein dnaA | 1795 | 52 kDa | 23 |
| large subunit ribosomal protein L23 | 1998 | 12 kDa | 14 |
| pyrophosphate-energized proton pump | 2044 | 75 kDa | 17 |
| ATP-dependent Clp protease proteolytic subunit | 262 | 23 kDa | 13 |
| exodeoxyribonuclease V | 812 | 141 kDa | 26 |
| predicted protein | 1177 | 99 kDa | 23 |
| glutamyl-tRNA synthetase | 1261 | 53 kDa | 24 |
| GTP-binding protein lepA | 1336 | 66 kDa | 21 |
| peptidyl-prolyl cis-trans iso | 122 | 18 kDa | 7 |
| large subunit ribosomal protein L20 | 151 | 13 kDa | 14 |
| signal peptidase I | 1335 | 35 kDa | 16 |
| histidinol-phosphate aminotransferase | 1891 | 40 kDa | 11 |
| transcription antiterminator | 1985 | 22 kDa | 10 |
| integration host factor subunit alpha | 154 | 15 kDa | 11 |
| exodeoxyribonuclease V | 813 | 126 kDa | 24 |
| phosphoglucosamine mutase | 1161 | 49 kDa | 20 |
| NAD-dependent epimerase/dehydratase | 1483 | 34 kDa | 13 |
| phosphoribosylformylglycinamidine cyclo-ligase | 1512 | 37 kDa | 12 |
| poly | 1516 | 53 kDa | 15 |
| 3-octaprenyl-4-hydroxybenzoate carboxy-lyase | 1584 | 56 kDa | 24 |
| UDP-N-acetylmuramoylalanine-D-glutamate ligase | 1652 | 54 kDa | 21 |
| preprotein translocase subunit SecF | 1849 | 35 kDa | 7 |
| peptide chain release factor 2 | 249 | 39 kDa | 16 |
| phosphate starvation-inducible protein | 405 | 32 kDa | 17 |
| two-component system sensor histidine kinase | 742 | 106 kDa | 27 |
| sensory box sensor/GGDEF/EAL domain-containing protein | 1310 | 123 kDa | 27 |
| DNA helicase II/ATP-dependent DNA helicase | 1631 | 85 kDa | 21 |
| glutathione synthetase | 1698 | 48 kDa | 18 |
| ABC transporter ATPase | 1209 | 74 kDa | 21 |
| peptidoglycan-associated lipoprotein | 1279 | 18 kDa | 13 |
| aminotransferase class-III | 1412 | 51 kDa | 18 |
| oligopeptidase A | 1614 | 78 kDa | 22 |
| imidazole glycerol phosphate synthase subunit HisH | 1889 | 24 kDa | 8 |
| predicted protein | 193 | 40 kDa | 14 |
| 2-dehydro-3-deoxyphosphooctonate aldolase | 928 | 31 kDa | 16 |
| diguanylate cyclase/phosphodiesterase with PAS/PAC and GAF sensor | 1119 | 114 kDa | 25 |
| tryptophan synthase subunit beta | 1345 | 45 kDa | 18 |
| radical SAM family protein | 1485 | 34 kDa | 19 |
| universal stress protein UspA | 1588 | 16 kDa | 10 |
| GTP-binding protein engA | 194 | 49 kDa | 14 |
| penicillin-binding protein 2 | 1303 | 73 kDa | 13 |
| GGDEF domain signalling protein | 1487 | 78 kDa | 16 |
| TPP-requiring enzyme | 1563 | 62 kDa | 18 |
| Zn-dependent hydrolase | 1830 | 23 kDa | 9 |
| stringent starvation protein A | 1881 | 24 kDa | 12 |
| diaminopimelate decarboxylase | 2026 | 47 kDa | 18 |
| glucose-6-phosphate isomerase | 140 | 61 kDa | 20 |
| tRNA modifying enzyme | 406 | 52 kDa | 18 |
| superoxide dismutase | 1322 | 22 kDa | 6 |
| exopolyphosphatase | 1389 | 54 kDa | 19 |
| transport-associated protein | 1910 | 23 kDa | 12 |
| ABC nitrate/sulfonate/bicarbonate family transporter | 359 | 35 kDa | 13 |
| quinolinate synthetase | 1113 | 41 kDa | 17 |
| conserved hypothetical protein | 1294 | 25 kDa | 11 |
| conserved hypothetical protein | 1381 | 44 kDa | 18 |
| D-amino acid dehydrogenase small subunit | 1617 | 46 kDa | 17 |
| predicted protein | 1685 | 52 kDa | 18 |
| universal stress protein family protein | 128 | 30 kDa | 14 |
| transglutaminase domain-containing protein | 447 | 24 kDa | 12 |
| conserved hypothetical protein | 498 | 37 kDa | 15 |
| predicted protein | 915 | 113 kDa | 20 |
| DNA-binding protein Hu-beta | 1221 | 10 kDa | 8 |
| tryptophan synthase subunit alpha | 1344 | 29 kDa | 13 |
| single-stranded DNA-binding protein | 1393 | 18 kDa | 10 |
| replicative DNA helicase | 1556 | 51 kDa | 16 |
| ADP-heptose synthase | 1630 | 34 kDa | 10 |
| UDP-N-acetylmuramate-alanine ligase | 1649 | 51 kDa | 16 |
| conserved hypothetical protein | 1678 | 151 kDa | 22 |
| tRNA nucleotidyltransferase polyA polymerase | 1914 | 47 kDa | 18 |
| PABA synthase | 1968 | 78 kDa | 19 |
| conserved hypothetical protein | 292 | 44 kDa | 16 |
| predicted protein | 902 | 37 kDa | 15 |
| DNA mismatch repair protein mutL | 1061 | 70 kDa | 20 |
| predicted protein | 1416 | 18 kDa | 13 |
| glycogen synthase | 1420 | 55 kDa | 18 |
| Sel1 repeat-containing protein | 592 | 33 kDa | 12 |
| 5-carboxymethyl-2-hydroxymuconate delta-isomerase | 788 | 31 kDa | 14 |
| iron-containing alcohol dehydrogenase | 792 | 42 kDa | 14 |
| conserved hypothetical protein | 925 | 18 kDa | 7 |
| L-aspartate oxidase | 938 | 60 kDa | 14 |
| thioredoxin reductase | 1133 | 34 kDa | 8 |
| two-component sensor histidine kinase | 1453 | 99 kDa | 18 |
| SSU ribosomal protein S16 | 1474 | 9 kDa | 7 |
| mitochondrial import inner membrane translocase | 1949 | 33 kDa | 10 |
| translation initiation factor IF-3 | 149 | 13 kDa | 14 |
| UDP-3-O-[3-hydroxymyristoyl] glucosamine N-acyltransferase | 276 | 37 kDa | 14 |
| heavy-metal transporting P-type ATPase | 493 | 91 kDa | 19 |
| protease | 983 | 51 kDa | 16 |
| conserved hypothetical protein | 1100 | 16 kDa | 7 |
| NAD+ synthase | 1128 | 60 kDa | 15 |
| holliday junction DNA helicase B | 1462 | 39 kDa | 14 |
| glutamate 5-kinase | 1530 | 40 kDa | 16 |
| carbohydrate kinase | 1578 | 34 kDa | 15 |
| orotidine 5'-phosphate decarboxylase | 2047 | 30 kDa | 13 |
| ATP/GTP binding protein | 98 | 18 kDa | 8 |
| outer membrane protein | 274 | 87 kDa | 19 |
| dipeptidase PepV | 364 | 52 kDa | 13 |
| predicted protein | 1066 | 38 kDa | 12 |
| phosphoribosylaminoimidazole-succinocarboxamide synthase | 1197 | 33 kDa | 13 |
| beta-hexosaminidase | 1329 | 39 kDa | 14 |
| Fe-S oxidoreductase | 1337 | 87 kDa | 22 |
| chorismate synthase | 1358 | 40 kDa | 10 |
| NADH-quinone oxidoreductase subunit G | 1366 | 85 kDa | 20 |
| exoribonuclease R | 1569 | 74 kDa | 16 |
| cytidylate kinase | 1625 | 25 kDa | 13 |
| dihydroorotase | 1809 | 39 kDa | 17 |
| dihydrodipicolinate reductase | 1974 | 29 kDa | 16 |
| LSU ribosomal protein L28P | 2075 | 9 kDa | 9 |
| RM-CspCI protein | 8 | 77 kDa | 14 |
| conserved hypothetical protein | 119 | 124 kDa | 22 |
| crispr-associated protein | 399 | 40 kDa | 15 |
| diguanylate cyclase/phosphodiesterase with PAS/PAC sensor | 956 | 116 kDa | 22 |
| alanine racemase | 966 | 39 kDa | 14 |
| molybdopterin biosynthesis MoeA protein | 1126 | 44 kDa | 15 |
| predicted protein | 1131 | 17 kDa | 8 |
| diguanylate cyclase/phosphodiesterase with PAS/PAC sensor | 1230 | 99 kDa | 15 |
| pyrimidine regulatory protein PyrR | 1289 | 19 kDa | 10 |
| GTP-binding protein Era | 1333 | 34 kDa | 15 |
| phosphate transport regulator | 1555 | 24 kDa | 12 |
| thiamine-phosphate pyrophosphorylase | 1633 | 24 kDa | 8 |
| GTP cyclohydrolase-2 | 1723 | 41 kDa | 13 |
| yecA family protein | 1760 | 25 kDa | 11 |
| ATP-dependent DNA helicase Rep | 1931 | 77 kDa | 20 |
| small subunit ribosomal protein S8 | 2010 | 14 kDa | 13 |
| penicillin-binding protein | 2028 | 82 kDa | 18 |
| conserved hypothetical protein | 118 | 121 kDa | 22 |
| lipid ABC transporter membrane/ATP-binding protein | 220 | 65 kDa | 14 |
| transcriptional activator NtrC | 241 | 56 kDa | 17 |
| HmsF protein | 890 | 81 kDa | 18 |
| DNA polymerase III subunit delta | 941 | 40 kDa | 15 |
| esterase | 1018 | 41 kDa | 12 |
| electron transfer flavoprotein alpha/beta-subunit | 1182 | 27 kDa | 11 |
| enoyl-[acyl-carrier-protein] reductase | 1256 | 29 kDa | 12 |
| ornithine carbamoyltransferase | 1667 | 35 kDa | 10 |
| nucleotide-binding protein | 1674 | 18 kDa | 12 |
| riboflavin synthase | 1753 | 22 kDa | 9 |
| GTP cyclohydrolase I | 1944 | 32 kDa | 11 |
| large subunit ribosomal protein L10 | 1988 | 18 kDa | 8 |
| conserved hypothetical protein | 243 | 59 kDa | 14 |
| conserved hypothetical protein | 631 | 144 kDa | 18 |
| conserved hypothetical protein | 1090 | 35 kDa | 13 |
| nicotinate-nucleotide pyrophosphorylase | 1112 | 31 kDa | 14 |
| glucose-1-phosphate adenylyltransferase | 1421 | 48 kDa | 13 |
| dihydroorotate dehydrogenase 2 | 1496 | 37 kDa | 13 |
| SWIB/MDM2 domain-containing protein | 1534 | 14 kDa | 13 |
| ABC-type transport system | 1898 | 24 kDa | 11 |
| lipoprotein nlpD | 158 | 29 kDa | 12 |
| uroporphyrin-III C-methyltransferase | 164 | 40 kDa | 16 |
| periplasmic serine protease | 186 | 52 kDa | 16 |
| conserved hypothetical protein | 192 | 24 kDa | 11 |
| signaling protein | 516 | 110 kDa | 16 |
| conserved hypothetical protein | 615 | 28 kDa | 7 |
| predicted protein | 674 | 17 kDa | 13 |
| Sel1 repeat-containing protein | 683 | 46 kDa | 17 |
| conserved hypothetical protein | 869 | 39 kDa | 12 |
| transcriptional repressor LexA | 1095 | 23 kDa | 13 |
| predicted protein | 1246 | 17 kDa | 10 |
| aspartate carbamoyltransferase | 1290 | 35 kDa | 15 |
| transcriptional regulator | 1550 | 34 kDa | 10 |
| peptidase M61 | 1781 | 69 kDa | 15 |
| imidazoleglycerol-phosphate dehydratase | 1890 | 23 kDa | 7 |
| nitroreductase | 1928 | 25 kDa | 9 |
| ribonucleotide-diphosphate reductase subunit beta | 19 | 43 kDa | 9 |
| conserved hypothetical protein | 230 | 18 kDa | 8 |
| beta-lactamase superfamily hydrolase | 287 | 31 kDa | 8 |
| peptidase M20C | 369 | 53 kDa | 14 |
| aminotransferase class I and II domain-containing protein | 525 | 46 kDa | 16 |
| cystathionine gamma-synthase | 526 | 43 kDa | 15 |
| predicted protein | 606 | 78 kDa | 13 |
| repressor protein cI | 626 | 24 kDa | 9 |
| predicted protein | 678 | 61 kDa | 20 |
| Mg-dependent DNase | 940 | 30 kDa | 16 |
| peptide chain release factor 1 | 1055 | 40 kDa | 17 |
| bacterioferritin comigratory protein | 1104 | 18 kDa | 8 |
| conserved hypothetical protein | 1105 | 63 kDa | 16 |
| transcription elongation factor protein | 1166 | 17 kDa | 10 |
| alcohol dehydrogenase GroES domain-containing protein | 1324 | 36 kDa | 9 |
| two component sensor histidine kinase transcription regulator protein | 1596 | 85 kDa | 16 |
| aldo/keto reductase | 1598 | 39 kDa | 14 |
| methenyltetrahydrofolate cyclohydrolase | 1613 | 31 kDa | 13 |
| drug efflux lipoprotein | 133 | 53 kDa | 11 |
| predicted protein | 422 | 192 kDa | 16 |
| ATP-dependent DNA helicase | 512 | 74 kDa | 20 |
| predicted protein | 637 | 68 kDa | 18 |
| conserved hypothetical protein | 672 | 89 kDa | 19 |
| ftsH protein | 732 | 72 kDa | 15 |
| 8-amino-7-oxononanoate synthase | 950 | 42 kDa | 15 |
| FAD/FMN-containing dehydrogenase | 955 | 51 kDa | 14 |
| plasma-membrane proton-efflux P-type ATPase | 1030 | 90 kDa | 15 |
| sorbitol dehydrogenase | 1069 | 38 kDa | 13 |
| conserved hypothetical protein | 1071 | 35 kDa | 11 |
| 30S ribosomal protein S18 | 1098 | 11 kDa | 8 |
| conserved hypothetical protein | 1165 | 17 kDa | 11 |
| molybdopterin/thiamine biosynthesis family protein | 1192 | 29 kDa | 10 |
| glycyl-tRNA synthetase | 1240 | 35 kDa | 11 |
| GatB/Yqey domain-containing protein | 1319 | 17 kDa | 12 |
| predicted protein | 1577 | 42 kDa | 9 |
| conserved hypothetical protein | 1839 | 10 kDa | 7 |
| riboflavin kinase | 49 | 36 kDa | 13 |
| predicted protein | 113 | 62 kDa | 15 |
| outer membrane autotransporter | 547 | 118 kDa | 12 |
| helix-turn-helix domain-containing protein | 647 | 39 kDa | 11 |
| molybdenum cofactor biosynthesis protein A | 894 | 42 kDa | 17 |
| AAA family ATPase | 1039 | 67 kDa | 18 |
| DNA primase | 1318 | 67 kDa | 14 |
| excinuclease ABC subunit C | 1328 | 72 kDa | 14 |
| polyphosphate kinase | 1387 | 86 kDa | 16 |
| guanosine-3',5'-bis | 1447 | 85 kDa | 13 |
| response regulator receiver:Metal-dependent phosphohydrolase | 1451 | 82 kDa | 15 |
| primosomal protein N' directs replication fork assembly at D-loops | 1586 | 76 kDa | 16 |
| S-adenosyl-methyltransferase MraW | 1658 | 35 kDa | 12 |
| 1-deoxy-D-xylulose-5-phosphate synthase | 1812 | 67 kDa | 15 |
| osmosensitive K+ channel signal transduction histidine kinase | 1874 | 99 kDa | 17 |
| N6-adenine-specific DNA methylase | 1969 | 44 kDa | 13 |
| ribosomal protein L33 | 2074 | 6 kDa | 6 |
| 50S ribosomal subunit protein L30 | 2014 | 7 kDa | 6 |
| conserved hypothetical protein | 1720 | 13 kDa | 5 |
| nitrogen regulatory protein PII | 1129 | 12 kDa | 4 |
| prephenate dehydrogenase/3-phosphoshikimate 1-carboxyvinyltransferase | 79 | 48 kDa | 12 |
| fatty acid/phospholipid synthesis protein plsX | 176 | 38 kDa | 11 |
| 3R-hydroxymyristoyl ACP dehydrase | 277 | 17 kDa | 8 |
| gp78 | 321 | 45 kDa | 14 |
| beta-lactamase domain-containing protein | 464 | 31 kDa | 11 |
| oxidoreductase iron-sulfur protein | 486 | 45 kDa | 13 |
| conserved hypothetical protein | 750 | 21 kDa | 8 |
| biotin synthesis protein bioC | 952 | 29 kDa | 7 |
| arabinose 5-phosphate isomerase | 1396 | 36 kDa | 10 |
| RNA polymerase factor sigma-54 | 1401 | 55 kDa | 14 |
| electron-transferring-flavoprotein dehydrogenase | 1425 | 61 kDa | 15 |
| peptidase M48 | 1499 | 30 kDa | 10 |
| aspartate 1-decarboxylase | 1543 | 15 kDa | 8 |
| predicted protein | 1579 | 10 kDa | 7 |
| phosphoglycolate phosphatase | 1592 | 25 kDa | 13 |
| dihydrolipoamide dehydrogenase | 1610 | 64 kDa | 13 |
| hydroxylamine reductase | 1765 | 60 kDa | 15 |
| integrase | 1776 | 49 kDa | 15 |
| ParB chromosome partitioning protein | 1804 | 32 kDa | 10 |
| lytic Murein transglycosylase | 1913 | 73 kDa | 16 |
| D-alanyl-D-alanine carboxypeptidase | 1983 | 52 kDa | 12 |
| Co-chaperone HscB | 33 | 21 kDa | 12 |
| UTP-glucose-1-phosphate uridylyltransferase | 205 | 33 kDa | 13 |
| membrane protease subunit | 285 | 34 kDa | 9 |
| ABC-type phosphate transport system | 412 | 34 kDa | 13 |
| predicted protein | 505 | 58 kDa | 14 |
| predicted protein | 796 | 13 kDa | 10 |
| DsrE/DsrF-like family protein | 874 | 13 kDa | 8 |
| Sel1 repeat-containing protein | 899 | 31 kDa | 15 |
| ssDNA exonuclease | 934 | 63 kDa | 12 |
| pyrroline-5-carboxylate reductase | 937 | 28 kDa | 11 |
| conserved hypothetical protein | 957 | 55 kDa | 14 |
| excinuclease ABC subunit B | 1084 | 80 kDa | 13 |
| conserved hypothetical protein | 1204 | 66 kDa | 12 |
| histone family protein nucleoid-structuring protein H-NS | 1236 | 11 kDa | 8 |
| formate dehydrogenase regulator | 1263 | 40 kDa | 12 |
| tol-Pal cell envelope complex subunit YbgF | 1278 | 27 kDa | 14 |
| conserved hypothetical protein | 1341 | 34 kDa | 11 |
| 3-deoxy-D-manno-octulosonate 8-phosphate phosphatase | 1397 | 19 kDa | 9 |
| competence lipoprotein ComL | 1444 | 31 kDa | 11 |
| glutathione S-transferase | 1540 | 23 kDa | 10 |
| Thiol:disulfide interchange protein dsbA | 1843 | 27 kDa | 11 |
| F0F1 ATP synthase subunit delta | 1826 | 19 kDa | 4 |
| RNase G | 227 | 55 kDa | 13 |
| glutamate decarboxylase | 246 | 52 kDa | 11 |
| membrane-associated metalloprotease | 273 | 50 kDa | 10 |
| two-component hybrid sensor kinase/response regulator | 389 | 60 kDa | 10 |
| aldo/keto reductase | 429 | 37 kDa | 10 |
| TPR repeat-containing protein | 559 | 33 kDa | 12 |
| conserved hypothetical protein | 598 | 45 kDa | 9 |
| two-component hybrid sensor kinase/response regulator | 648 | 63 kDa | 19 |
| bacteriophage protein | 711 | 116 kDa | 6 |
| alcohol dehydrogenase | 838 | 37 kDa | 9 |
| methyltransferase type 11 | 922 | 30 kDa | 10 |
| dTDP-6-deoxy-D-xylo-hex-3-ulose aminase | 1065 | 41 kDa | 14 |
| myo-inositol-1 | 1093 | 29 kDa | 8 |
| aminotransferase | 1202 | 44 kDa | 10 |
| conserved hypothetical protein | 1214 | 17 kDa | 8 |
| ribonuclease III | 1334 | 31 kDa | 10 |
| conserved hypothetical protein | 1786 | 29 kDa | 6 |
| conserved hypothetical protein | 1811 | 30 kDa | 11 |
| regulatory protein | 1952 | 9 kDa | 7 |
| Zn-dependent peptidase | 2061 | 50 kDa | 15 |
| virulence factor MviN | 1526 | 56 kDa | 4 |
| conserved hypothetical protein | 6 | 34 kDa | 11 |
| HflX GTP-binding protein | 196 | 42 kDa | 11 |
| conserved hypothetical protein | 286 | 12 kDa | 8 |
| deoxyribodipyrimidine photolyase | 351 | 58 kDa | 14 |
| outer membrane porin protein | 365 | 40 kDa | 9 |
| autotransporter | 485 | 90 kDa | 17 |
| TPR repeat-containing protein | 553 | 24 kDa | 9 |
| conserved hypothetical protein | 595 | 56 kDa | 11 |
| iron-containing alcohol dehydrogenase | 597 | 44 kDa | 13 |
| conserved hypothetical protein | 726 | 53 kDa | 14 |
| tRNA-dihydrouridine synthase A | 731 | 37 kDa | 12 |
| GGDEF domain-containing protein | 851 | 51 kDa | 12 |
| predicted protein | 901 | 17 kDa | 6 |
| phytoene synthase | 960 | 32 kDa | 8 |
| HlyD family secretion protein | 990 | 33 kDa | 14 |
| XRE family transcriptional regulator | 1079 | 22 kDa | 8 |
| conserved hypothetical protein | 1227 | 18 kDa | 8 |
| membrane-bound lytic Murein transglycosylase D | 1255 | 59 kDa | 14 |
| N-acetylglucosaminyl transferase | 1629 | 45 kDa | 10 |
| UDP-N-acetylmuramyl pentapeptide synthase | 1654 | 50 kDa | 16 |
| multiphosphoryl transfer protein 1 | 1695 | 10 kDa | 9 |
| radical SAM domain-containing protein | 1748 | 23 kDa | 10 |
| conserved hypothetical protein | 1752 | 57 kDa | 15 |
| resistance protein | 1759 | 15 kDa | 7 |
| peptide chain release factor RF-3 | 1923 | 63 kDa | 13 |
| GTP-dependent nucleic acid-binding protein EngD | 2067 | 40 kDa | 11 |
| predicted protein | 2071 | 21 kDa | 10 |
| ATP-dependent protease peptidase subunit | 1930 | 19 kDa | 6 |
| F0F1 ATP synthase subunit C | 1828 | 8 kDa | 2 |
| heme-binding protein A | 50 | 65 kDa | 15 |
| conserved hypothetical protein | 62 | 53 kDa | 13 |
| diaminopimelate epimerase | 69 | 31 kDa | 11 |
| cys regulon transcriptionnal regulator protein | 93 | 35 kDa | 14 |
| NADPH2:quinone reductase | 109 | 35 kDa | 14 |
| conserved hypothetical protein | 139 | 57 kDa | 12 |
| membrane protease subunit HflK | 197 | 47 kDa | 13 |
| glutaminase GlsA | 245 | 34 kDa | 13 |
| MscS mechanosensitive ion channel | 370 | 49 kDa | 16 |
| conserved hypothetical protein | 463 | 86 kDa | 10 |
| heavy metal translocating P-type ATPase | 530 | 67 kDa | 13 |
| predicted protein | 632 | 78 kDa | 18 |
| Sel1 repeat-containing protein | 653 | 59 kDa | 10 |
| 3,4-dihydroxy-2-butanone 4-phosphate synthase | 735 | 24 kDa | 8 |
| transcriptional activator MetR | 1032 | 34 kDa | 9 |
| conserved hypothetical protein | 1111 | 65 kDa | 19 |
| metallo-beta-lactamase superfamily protein | 1149 | 36 kDa | 11 |
| cystathionine beta-lyase | 1440 | 43 kDa | 9 |
| carbonic anhydrase 2 | 1521 | 27 kDa | 9 |
| conserved hypothetical protein | 1582 | 12 kDa | 6 |
| glutathione synthetase | 1697 | 35 kDa | 9 |
| conserved hypothetical protein | 1965 | 23 kDa | 9 |
| cell division ATP-binding protein FtsE | 2059 | 25 kDa | 6 |
| thioredoxin | 132 | 12 kDa | 6 |
| two-domain glycosyltransferase | 224 | 31 kDa | 9 |
| conserved hypothetical protein | 354 | 87 kDa | 14 |
| rnfC protein | 508 | 46 kDa | 11 |
| predicted protein | 536 | 22 kDa | 6 |
| cation transport ATPase | 780 | 107 kDa | 12 |
| D-isomer specific 2-hydroxyacid dehydrogenase | 849 | 39 kDa | 11 |
| ribosomal large subunit pseudouridine synthase C | 887 | 38 kDa | 8 |
| Sel1 repeat-containing protein | 935 | 61 kDa | 14 |
| uracil DNA glycosylase | 1083 | 30 kDa | 6 |
| branched-chain amino acid aminotransferase | 1201 | 34 kDa | 6 |
| predicted protein | 1281 | 33 kDa | 9 |
| GTPase | 1299 | 38 kDa | 8 |
| recombination factor protein RarA | 1430 | 48 kDa | 14 |
| DNA-directed RNA polymerase subunit omega | 1446 | 8 kDa | 10 |
| UDP-N-acetylglucosamine-N-acetylmuramyl- | 1650 | 39 kDa | 13 |
| translation initiation factor 2B subunit I family | 1672 | 38 kDa | 11 |
| anthranilate phosphoribosyltransferase | 1783 | 37 kDa | 8 |
| ribonuclease P protein component | 1793 | 15 kDa | 12 |
| geranyltranstransferase | 1813 | 32 kDa | 8 |
| lipoyl synthase | 1841 | 36 kDa | 14 |
| imidazole glycerol phosphate synthase subunit hisF | 1887 | 27 kDa | 9 |
| heme-binding protein A | 1956 | 66 kDa | 14 |
| pmbA | 1966 | 48 kDa | 10 |
| deoxyguanosinetriphosphate triphosphohydrolase | 2030 | 44 kDa | 12 |
| predicted protein | 795 | 10 kDa | 5 |
| iron-sulfur cluster insertion protein ErpA | 38 | 13 kDa | 4 |
| rubrerythrin | 130 | 16 kDa | 10 |
| conserved hypothetical protein | 198 | 33 kDa | 13 |
| pseudouridylate synthase | 207 | 28 kDa | 14 |
| acyl-[acyl-carrier-protein]-UDP-N-acetylglucosamine O-acyltransferase | 278 | 28 kDa | 9 |
| autotransporter protein | 347 | 293 kDa | 11 |
| nitroreductase | 378 | 21 kDa | 6 |
| succinate-semialdehyde dehydrogenase | 540 | 53 kDa | 8 |
| conserved hypothetical protein | 727 | 55 kDa | 11 |
| exonuclease family | 881 | 37 kDa | 14 |
| conserved hypothetical protein | 1008 | 22 kDa | 9 |
| copper-transporting ATPase | 1027 | 76 kDa | 15 |
| succinyl-diaminopimelate desuccinylase | 1207 | 41 kDa | 8 |
| conserved hypothetical protein | 1260 | 46 kDa | 10 |
| bifunctional folylpolyglutamate synthase/dihydrofolate synthase | 1342 | 48 kDa | 12 |
| 3-isopropylmalate dehydratase small subunit | 1355 | 24 kDa | 8 |
| acetyltransferase | 1433 | 21 kDa | 10 |
| two-component system sensor histidine kinase | 1489 | 94 kDa | 14 |
| ribosome-associated GTPase | 1666 | 33 kDa | 9 |
| predicted protein | 1683 | 14 kDa | 9 |
| prophage Lp2 protein 4 | 1768 | 55 kDa | 12 |
| acetylglutamate kinase | 1821 | 32 kDa | 8 |
| potassium-transporting ATPase subunit B | 1876 | 73 kDa | 8 |
| exodeoxyribonuclease VII large subunit | 2031 | 49 kDa | 11 |
| biopolymer transporter ExbB | 2038 | 23 kDa | 5 |
| RNA polymerase sigma-32 factor | 2057 | 34 kDa | 8 |
| signal recognition particle receptor | 2060 | 38 kDa | 10 |
| two-components system response regulator | 1597 | 26 kDa | 6 |
| predicted protein | 1603 | 19 kDa | 5 |
| phosphoglycolate phosphatase | 72 | 24 kDa | 11 |
| molybdopterin binding protein | 238 | 29 kDa | 10 |
| conserved hypothetical protein | 265 | 54 kDa | 9 |
| conserved hypothetical protein | 288 | 13 kDa | 6 |
| conserved hypothetical protein | 379 | 63 kDa | 13 |
| conserved hypothetical protein | 430 | 68 kDa | 11 |
| predicted protein | 465 | 51 kDa | 8 |
| transcriptional regulator containing GAF | 488 | 59 kDa | 12 |
| conserved hypothetical protein | 515 | 59 kDa | 12 |
| YgiW protein | 571 | 14 kDa | 6 |
| Sel1 repeat-containing protein | 819 | 35 kDa | 10 |
| amino acid racemase | 821 | 40 kDa | 11 |
| DNA polymerase III subunit epsilon | 982 | 27 kDa | 8 |
| outer membrane efflux protein | 991 | 52 kDa | 9 |
| coproporphyrinogen III oxidase | 1044 | 48 kDa | 11 |
| ribonuclease PH | 1082 | 27 kDa | 10 |
| vitamin B12-binding protein | 1269 | 31 kDa | 8 |
| MreC rod shape-determining protein | 1305 | 33 kDa | 10 |
| NADH-ubiquinone oxidoreductase D subunit | 1369 | 48 kDa | 15 |
| conserved hypothetical protein | 1449 | 52 kDa | 12 |
| conserved hypothetical protein | 1494 | 16 kDa | 8 |
| 4Fe-4S cluster binding | 1506 | 43 kDa | 11 |
| 2-hydroxy-3-oxopropionate reductase | 1537 | 31 kDa | 10 |
| integration host factor subunit beta | 1627 | 11 kDa | 7 |
| FtsI cell division protein | 1656 | 64 kDa | 12 |
| DNA-binding response regulator | 1663 | 26 kDa | 14 |
| glycerol-3-phosphate dehydrogenase | 1680 | 35 kDa | 8 |
| conserved hypothetical protein | 1817 | 28 kDa | 9 |
| predicted protein | 1831 | 25 kDa | 10 |
| ABC-type transport system | 1900 | 16 kDa | 9 |
| predicted protein | 1905 | 88 kDa | 13 |
| GGDEF domain-containing protein | 2045 | 62 kDa | 14 |
| phosphopantetheine adenylyltransferase | 2064 | 19 kDa | 8 |
| conserved hypothetical protein | 814 | 14 kDa | 5 |
| phosphoglycolate phosphatase | 886 | 24 kDa | 5 |
| iron-binding protein iscA | 34 | 12 kDa | 4 |
| 50S ribosomal subunit protein A | 150 | 8 kDa | 4 |
| ATP-dependent DNA helicase | 15 | 69 kDa | 15 |
| lipid A biosynthesis lauroyl acyltransferase | 70 | 34 kDa | 10 |
| amino-acid N-acetyltransferase | 95 | 49 kDa | 9 |
| phosphatidylserine decarboxylase subunit proenzyme | 102 | 24 kDa | 9 |
| D-alanyl-D-alanine-endopeptidase | 201 | 43 kDa | 11 |
| DNA-directed DNA polymerase | 356 | 48 kDa | 14 |
| conserved hypothetical protein | 401 | 47 kDa | 10 |
| conserved hypothetical protein | 403 | 82 kDa | 16 |
| drug efflux pump transmembrane protein | 471 | 113 kDa | 12 |
| dihydropyrimidine dehydrogenase | 487 | 43 kDa | 15 |
| acyltransferase 3 | 510 | 78 kDa | 10 |
| two-component hybrid sensor kinase/response regulator | 529 | 90 kDa | 11 |
| conserved hypothetical protein | 542 | 18 kDa | 9 |
| conserved hypothetical protein | 825 | 60 kDa | 10 |
| MFS family transporter | 953 | 59 kDa | 8 |
| predicted protein | 1067 | 49 kDa | 12 |
| Muramoyltetrapeptide carboxypeptidase | 1115 | 36 kDa | 8 |
| conserved hypothetical protein | 1147 | 100 kDa | 12 |
| conserved hypothetical protein | 1176 | 34 kDa | 9 |
| signal transduction protein | 1212 | 80 kDa | 13 |
| nucleotidyl transferase | 1544 | 27 kDa | 6 |
| membrane-bound lytic Murein transglycosylase | 1589 | 44 kDa | 11 |
| L-fuculose-phosphate aldolase | 1670 | 25 kDa | 6 |
| glutaredoxin 3 | 1682 | 10 kDa | 9 |
| conserved hypothetical protein | 1710 | 135 kDa | 7 |
| queuosine biosynthesis protein queC | 1712 | 28 kDa | 8 |
| 4-alpha-glucanotransferase | 1854 | 58 kDa | 10 |
| CTP:CMP-3-deoxy-D-manno-octulosonate transferase | 2042 | 28 kDa | 8 |
| oxidoreductase | 1604 | 31 kDa | 5 |
| histidine triad protein | 1884 | 13 kDa | 4 |
| predicted protein | 1379 | 9 kDa | 3 |
| cold-shock DNA-binding domain-containing protein | 1608 | 7 kDa | 3 |
| ModE family transcriptional regulator | 59 | 27 kDa | 9 |
| ubiquinone biosynthesis O-methyltransferase | 73 | 26 kDa | 8 |
| ATPase | 137 | 94 kDa | 12 |
| toxin ABC transporter ATPase and permease | 143 | 80 kDa | 9 |
| 2-dehydropantoate 2-reductase | 169 | 36 kDa | 8 |
| predicted protein | 331 | 97 kDa | 12 |
| conserved hypothetical protein | 363 | 52 kDa | 11 |
| HipA domain-containing protein | 481 | 46 kDa | 11 |
| conserved hypothetical protein | 499 | 113 kDa | 11 |
| Sel1 repeat-containing protein | 532 | 57 kDa | 10 |
| conserved hypothetical protein | 552 | 28 kDa | 9 |
| methyltransferase FkbM family | 593 | 27 kDa | 8 |
| endodeoxyribonuclease RusA | 630 | 25 kDa | 9 |
| UspA protein | 781 | 34 kDa | 5 |
| phosphatidylinositol phospholipase C | 875 | 41 kDa | 10 |
| ankyrin repeat-containing signal peptide protein | 939 | 27 kDa | 11 |
| large subunit ribosomal protein L21 | 1527 | 12 kDa | 7 |
| adenosylmethionine-8-amino-7-oxononanoate transaminase | 1619 | 50 kDa | 12 |
| predicted protein | 1702 | 23 kDa | 7 |
| histone deacetylase | 1718 | 34 kDa | 9 |
| DNA repair protein RecN | 2050 | 61 kDa | 12 |
| conserved hypothetical protein | 2062 | 49 kDa | 12 |
| deoxycytidine triphosphate deaminase | 1286 | 21 kDa | 5 |
| hydroxyethylthiazole kinase | 64 | 29 kDa | 5 |
| carboxylesterase | 1963 | 24 kDa | 3 |
| deoxyuridine 5'-triphosphate nucleotidohydrolase | 45 | 16 kDa | 3 |
| molybdenum import ATP-binding protein ModC | 56 | 39 kDa | 10 |
| UDP-2,3-diacylglucosamine hydrolase | 121 | 29 kDa | 10 |
| glycosyl transferase family 2 protein | 222 | 29 kDa | 9 |
| conserved hypothetical protein | 282 | 31 kDa | 9 |
| nuclease domain-containing protein | 432 | 22 kDa | 8 |
| conserved hypothetical protein | 458 | 26 kDa | 9 |
| conserved hypothetical protein | 569 | 35 kDa | 8 |
| predicted protein | 572 | 25 kDa | 7 |
| predicted protein | 671 | 30 kDa | 11 |
| diguanylate cyclase | 836 | 56 kDa | 10 |
| type I pili usher protein CsuD | 911 | 86 kDa | 12 |
| UDP-glcNAc-lipooligosaccharide N-acetylglucosamine glycosyltransferase | 1064 | 38 kDa | 12 |
| GGDEF family protein | 1434 | 89 kDa | 10 |
| conserved hypothetical protein | 1442 | 27 kDa | 7 |
| O-sialoglycoprotein endopeptidase | 1477 | 36 kDa | 6 |
| OstA organic solvent tolerance protein | 1546 | 85 kDa | 12 |
| 3-deoxy-D-manno-octulosonic-acid transferase | 1662 | 47 kDa | 13 |
| thiamine biosynthesis protein ThiF | 1687 | 28 kDa | 8 |
| short chain dehydrogenase | 1704 | 28 kDa | 8 |
| thiol:disulfide interchange protein DsbC | 1778 | 28 kDa | 9 |
| ABC transporter ATP-binding protein | 1960 | 64 kDa | 10 |
| pirin family protein | 893 | 26 kDa | 6 |
| predicted protein | 970 | 29 kDa | 5 |
| phosphoribosylformimino-5-aminoimidazole carboxamide isomerase | 1888 | 27 kDa | 5 |
| Polyphosphate:AMP phosphotransferase | 68 | 34 kDa | 7 |
| type I secretion outer membrane protein | 147 | 57 kDa | 8 |
| transcriptional regulator | 170 | 33 kDa | 9 |
| conserved hypothetical protein | 213 | 12 kDa | 9 |
| predicted protein | 393 | 35 kDa | 11 |
| predicted protein | 483 | 34 kDa | 7 |
| Sel1 repeat-containing protein | 539 | 45 kDa | 7 |
| outer membrane porin protein | 575 | 40 kDa | 7 |
| conserved hypothetical protein | 677 | 54 kDa | 13 |
| extended-spectrum class A beta lactamase | 688 | 34 kDa | 10 |
| predicted protein | 884 | 17 kDa | 7 |
| glutamyl-tRNA synthetase | 980 | 33 kDa | 7 |
| TonB-dependent receptor plug | 1050 | 86 kDa | 12 |
| molybdopterin-converting factor subunit 2 | 1110 | 17 kDa | 6 |
| conserved hypothetical protein | 1160 | 86 kDa | 10 |
| hydrolase | 1174 | 33 kDa | 10 |
| phosphoribosylaminoimidazole carboxylase catalytic subunit | 1198 | 18 kDa | 8 |
| ribosome-binding factor A | 1224 | 14 kDa | 7 |
| triosephosphate isomerase | 1377 | 27 kDa | 11 |
| conserved hypothetical protein | 1398 | 23 kDa | 8 |
| choloylglycine hydrolase | 1414 | 41 kDa | 5 |
| UDP-3-O-[3-hydroxymyristoyl] N-acetylglucosamine deacetylase | 1644 | 35 kDa | 8 |
| D-alanine-D-alanine ligase | 1648 | 35 kDa | 9 |
| oligoribonuclease | 1664 | 23 kDa | 9 |
| S-adenosylmethionine:tRNA ribosyltransferase-isomerase | 1853 | 39 kDa | 12 |
| conserved hypothetical protein | 310 | 31 kDa | 6 |
| preprotein translocase SecY subunit | 2016 | 48 kDa | 6 |
| predicted protein | 89 | 10 kDa | 4 |
| MesJ cell cycle protein | 126 | 54 kDa | 10 |
| HemY protein | 165 | 46 kDa | 7 |
| PII uridylyltransferase | 208 | 99 kDa | 10 |
| HicB family protein | 316 | 12 kDa | 7 |
| conserved hypothetical protein | 320 | 62 kDa | 8 |
| outer membrane autotransporter | 433 | 145 kDa | 11 |
| outer membrane autotransporter barrel domain-containing protein | 452 | 84 kDa | 10 |
| Sel1 repeat-containing protein | 504 | 34 kDa | 12 |
| short-chain dehydrogenase/reductase SDR | 537 | 31 kDa | 8 |
| thymidylate synthetase | 538 | 31 kDa | 6 |
| nitroreductase | 548 | 32 kDa | 10 |
| predicted protein | 551 | 37 kDa | 9 |
| predicted protein | 558 | 12 kDa | 7 |
| two-component hybrid sensor kinase/response regulator | 728 | 77 kDa | 10 |
| conserved hypothetical protein | 729 | 12 kDa | 8 |
| predicted protein | 834 | 25 kDa | 8 |
| peptidase U7 family | 885 | 35 kDa | 7 |
| predicted protein | 903 | 37 kDa | 8 |
| ATPase | 958 | 80 kDa | 8 |
| predicted protein | 1141 | 21 kDa | 6 |
| inorganic diphosphatase | 1152 | 38 kDa | 9 |
| conserved hypothetical protein | 1234 | 30 kDa | 8 |
| peptidase M48 family | 1258 | 54 kDa | 8 |
| glycogen branching enzyme | 1424 | 83 kDa | 10 |
| acetyl-hydrolase | 1490 | 42 kDa | 7 |
| L-alanyl-gamma-D-glutamyl-meso-diaminopimelate ligase | 1571 | 51 kDa | 7 |
| nitrogen fixation protein VnfA | 1605 | 53 kDa | 7 |
| anaerobic ribonucleoside-triphosphate reductase | 1745 | 8 kDa | 8 |
| indole-3-glycerol-phosphate synthase | 1782 | 29 kDa | 8 |
| dephospho-CoA kinase | 1787 | 23 kDa | 8 |
| ABC transport system ATP-binding protein | 1834 | 29 kDa | 9 |
| conserved hypothetical protein | 1878 | 82 kDa | 9 |
| lipopolysaccharide heptosyltransferase I | 1911 | 37 kDa | 8 |
| nitrilase | 1677 | 29 kDa | 6 |
| transcriptional regulator | 1767 | 16 kDa | 6 |
| Sigma | 1402 | 13 kDa | 5 |
| nicotinic acid mononucleotide adenylyltransferase | 231 | 25 kDa | 4 |
| predicted protein | 1376 | 13 kDa | 4 |
| conserved hypothetical protein | 1844 | 21 kDa | 4 |
| transcriptional regulator | 1789 | 22 kDa | 3 |
| SurE survival protein | 156 | 27 kDa | 7 |
| response regulator receiver protein | 414 | 26 kDa | 7 |
| deoxyguanosinetriphosphate triphosphohydrolase | 599 | 51 kDa | 11 |
| conserved hypothetical protein | 778 | 51 kDa | 8 |
| Ku domain-containing protein | 801 | 30 kDa | 8 |
| predicted protein | 807 | 39 kDa | 8 |
| exodeoxyribonuclease V | 811 | 68 kDa | 12 |
| conserved hypothetical protein | 835 | 32 kDa | 8 |
| transcriptional regulator | 871 | 34 kDa | 9 |
| phytase | 876 | 42 kDa | 11 |
| thymidylate kinase | 942 | 24 kDa | 7 |
| methyltransferase | 1022 | 27 kDa | 9 |
| beta-lactamase domain-containing protein | 1038 | 35 kDa | 10 |
| predicted protein | 1051 | 35 kDa | 9 |
| dTDP-D-glucose 4,6-dehydratase | 1075 | 39 kDa | 9 |
| glycogen debranching enzyme | 1423 | 77 kDa | 12 |
| outer membrane autotransporter barrel domain-containing protein | 1431 | 181 kDa | 12 |
| molecular chaperone | 1450 | 40 kDa | 8 |
| monofunctional biosynthetic peptidoglycan transglycosylase | 1566 | 28 kDa | 6 |
| cysteine synthase | 1580 | 33 kDa | 7 |
| RNA pseudouridine synthase | 1609 | 28 kDa | 9 |
| methylthioadenosine phosphorylase | 1669 | 32 kDa | 6 |
| phosphoglyceromutase 1 | 1684 | 28 kDa | 9 |
| homoserine O-acetyltransferase | 1693 | 42 kDa | 6 |
| competence protein comM | 1700 | 55 kDa | 10 |
| DNA-methyltransferase | 1769 | 33 kDa | 6 |
| penicillin-binding protein 6. serine peptidase. merops family s11 | 1838 | 44 kDa | 8 |
| ATP-dependent DNA helicase recG | 1855 | 76 kDa | 14 |
| peptidase S1 and S6 | 1883 | 41 kDa | 9 |
| uroporphyrin-III C/tetrapyrrole methyltransferase | 1907 | 33 kDa | 5 |
| conserved hypothetical protein | 1917 | 39 kDa | 6 |
| 2-polyprenylphenol 6-hydroxylase | 1950 | 60 kDa | 9 |
| heat shock protein HtpX | 1172 | 32 kDa | 5 |
| major facilitator transporter | 489 | 46 kDa | 6 |
| ApbE family lipoprotein | 507 | 34 kDa | 5 |
| D-tyrosyl-tRNA | 1454 | 16 kDa | 4 |
| export protein secB | 1681 | 17 kDa | 4 |
| predicted protein | 2058 | 34 kDa | 4 |
| biotin- | 1740 | 26 kDa | 4 |
| predicted protein | 913 | 10 kDa | 3 |
| acyl carrier protein | 180 | 9 kDa | 2 |
| macrolide export ATP-binding/permease macB | 12 | 70 kDa | 9 |
| regulatory protein TtgR | 136 | 24 kDa | 7 |
| secretion system protein D | 142 | 44 kDa | 7 |
| conserved hypothetical protein | 184 | 33 kDa | 6 |
| 4-hydroxy-3-methylbut-2-en-1-yl diphosphate synthase | 190 | 46 kDa | 10 |
| conserved hypothetical protein | 311 | 26 kDa | 7 |
| helix-turn-helix domain-containing protein | 343 | 11 kDa | 8 |
| predicted protein | 431 | 33 kDa | 10 |
| RND efflux system outer membrane lipoprotein | 470 | 58 kDa | 9 |
| drug efflux pump transmembrane protein | 472 | 112 kDa | 9 |
| conserved hypothetical protein | 497 | 39 kDa | 8 |
| Sel1 repeat-containing protein | 523 | 33 kDa | 8 |
| predicted protein | 524 | 63 kDa | 9 |
| predicted protein | 535 | 121 kDa | 8 |
| predicted protein | 557 | 53 kDa | 10 |
| conserved hypothetical protein | 565 | 44 kDa | 10 |
| phospholipid-lipopolysaccharide ABC transporter | 570 | 64 kDa | 7 |
| TPR repeat-containing protein | 590 | 33 kDa | 9 |
| magnesium and cobalt efflux protein CorC | 614 | 33 kDa | 10 |
| predicted protein | 832 | 26 kDa | 6 |
| Sel1 repeat-containing protein | 863 | 30 kDa | 11 |
| hydrophobe/amphiphile efflux-1 family protein | 947 | 117 kDa | 10 |
| DNA polymerase bacteriophage-type | 974 | 34 kDa | 5 |
| CoA-binding protein | 978 | 15 kDa | 9 |
| p-Nitrobenzoate reductase | 1011 | 26 kDa | 9 |
| tRNA delta | 1063 | 35 kDa | 11 |
| succinyldiaminopimelate aminotransferase | 1205 | 46 kDa | 6 |
| ABC transporter ATP-binding protein | 1400 | 27 kDa | 8 |
| ADP-ribose pyrophosphatase | 1429 | 23 kDa | 7 |
| resolvase, N-terminal:Recombinase | 1492 | 29 kDa | 10 |
| conserved hypothetical protein | 1503 | 37 kDa | 7 |
| methylated-DNA-[protein]-cysteine methyltransferase | 1505 | 18 kDa | 8 |
| dimethyladenosine transferase dimethyltransferase | 1549 | 29 kDa | 6 |
| thiamine-monophosphate kinase | 1558 | 34 kDa | 7 |
| ribosomal protein L11 methyltransferase | 1576 | 33 kDa | 6 |
| conserved hypothetical protein | 1623 | 11 kDa | 7 |
| UDP-N-acetylmuramate dehydrogenase | 1673 | 38 kDa | 8 |
| conserved hypothetical protein | 1810 | 20 kDa | 10 |
| homoserine kinase | 1819 | 37 kDa | 6 |
| peptidyl-tRNA hydrolase | 1934 | 22 kDa | 6 |
| predicted protein | 1939 | 24 kDa | 6 |
| rare lipoprotein B | 1972 | 23 kDa | 8 |
| endonuclease/exonuclease/phosphatase | 1661 | 30 kDa | 5 |
| conserved hypothetical protein | 1837 | 22 kDa | 5 |
| conserved hypothetical protein | 300 | 24 kDa | 4 |
| conserved hypothetical protein | 1195 | 16 kDa | 5 |
| conserved hypothetical protein | 771 | 18 kDa | 4 |
| glyoxalase I | 1245 | 15 kDa | 3 |
| small subunit ribosomal protein S19 | 2000 | 6 kDa | 3 |
| RNA pseudouridylate synthase | 2 | 37 kDa | 8 |
| RNA polymerase factor sigma-70 | 24 | 21 kDa | 7 |
| conserved hypothetical protein | 63 | 24 kDa | 5 |
| conserved hypothetical protein | 299 | 24 kDa | 7 |
| predicted protein | 304 | 32 kDa | 8 |
| ClpP protease | 322 | 29 kDa | 9 |
| predicted protein | 328 | 14 kDa | 6 |
| two-component sensor | 415 | 50 kDa | 7 |
| outer membrane autotransporter | 419 | 72 kDa | 8 |
| predicted protein | 444 | 30 kDa | 11 |
| pyruvate dehydrogenase | 461 | 62 kDa | 6 |
| HAD family hydrolase | 514 | 27 kDa | 8 |
| TPR repeat-containing protein | 556 | 34 kDa | 7 |
| TPR repeat-containing protein | 560 | 12 kDa | 5 |
| conserved hypothetical protein | 588 | 78 kDa | 10 |
| outer membrane autotransporter | 618 | 101 kDa | 10 |
| Sel1 repeat-containing protein | 643 | 52 kDa | 10 |
| predicted protein | 652 | 52 kDa | 7 |
| conserved hypothetical protein | 743 | 18 kDa | 6 |
| predicted protein | 748 | 19 kDa | 6 |
| predicted protein | 790 | 9 kDa | 6 |
| triphosphoribosyl-dephospho-CoA transferase | 826 | 31 kDa | 9 |
| short-chain dehydrogenase/reductase SDR | 846 | 26 kDa | 6 |
| predicted protein | 859 | 137 kDa | 6 |
| HipA domain-containing protein | 898 | 48 kDa | 8 |
| predicted protein | 914 | 31 kDa | 10 |
| predicted protein | 1000 | 52 kDa | 9 |
| conserved hypothetical protein | 1029 | 32 kDa | 6 |
| transcriptional regulator | 1035 | 50 kDa | 8 |
| dTDP-4-dehydrorhamnose reductase | 1076 | 31 kDa | 7 |
| coproporphyrinogen oxidase | 1080 | 46 kDa | 11 |
| peptidase | 1144 | 33 kDa | 8 |
| dihydropteroate synthase | 1162 | 30 kDa | 6 |
| translation factor | 1171 | 23 kDa | 5 |
| signal transduction protein | 1213 | 66 kDa | 7 |
| tRNA pseudouridine synthase A | 1347 | 30 kDa | 7 |
| tRNA-dihydrouridine synthase | 1457 | 36 kDa | 8 |
| N-acetylmuramoyl-L-alanine amidase | 1508 | 48 kDa | 10 |
| conserved hypothetical protein | 1531 | 20 kDa | 8 |
| dinucleoside polyphosphate hydrolase | 1532 | 24 kDa | 8 |
| tRNA-specific 2-thiouridylase mnmA | 1541 | 41 kDa | 9 |
| magnesium and cobalt transporter CorA | 1565 | 37 kDa | 9 |
| DNA processing protein | 1600 | 41 kDa | 8 |
| peptide deformylase | 1601 | 23 kDa | 7 |
| peptidase M24 | 1802 | 68 kDa | 6 |
| ABC transport system ATP-binding protein | 1902 | 30 kDa | 7 |
| histone acetyltransferase HPA2 | 1915 | 25 kDa | 10 |
| mog molybdopterin biosynthesis protein | 1964 | 23 kDa | 8 |
| GTP-binding protein EngB | 2025 | 26 kDa | 6 |
| biopolymer transporter | 2039 | 16 kDa | 8 |
| smr protein/MutS2 | 1132 | 24 kDa | 5 |
| conserved hypothetical protein | 97 | 20 kDa | 5 |
| gp9 | 326 | 12 kDa | 5 |
| 3-dehydroquinate dehydratase II | 1573 | 16 kDa | 5 |
| riboflavin synthase subunit beta | 1724 | 18 kDa | 5 |
| inorganic polyphosphate/NAD+ kinase | 2049 | 32 kDa | 4 |
| predicted protein | 794 | 11 kDa | 3 |
| early gene regulator | 2076 | 14 kDa | 4 |
| conserved hypothetical protein | 1514 | 26 kDa | 3 |
| acetyl-CoA carboxylase | 1574 | 17 kDa | 3 |
| serine O-acetyltransferase | 117 | 27 kDa | 9 |
| ADP-heptose:LPS heptosyltransferase II | 221 | 39 kDa | 6 |
| amino acid transporters | 244 | 57 kDa | 6 |
| exodeoxyribonuclease III | 251 | 29 kDa | 5 |
| HNH endonuclease | 318 | 13 kDa | 6 |
| conserved hypothetical protein | 345 | 45 kDa | 7 |
| phosphate uptake regulator | 413 | 28 kDa | 7 |
| predicted protein | 423 | 17 kDa | 6 |
| pentapeptide repeat-containing protein | 446 | 22 kDa | 6 |
| outer membrane autotransporter barrel domain-containing protein | 583 | 165 kDa | 7 |
| phage Tail Collar Domain containing protein | 646 | 29 kDa | 6 |
| conserved hypothetical protein | 673 | 42 kDa | 7 |
| conserved hypothetical protein | 687 | 30 kDa | 7 |
| aldo/keto reductase | 848 | 37 kDa | 9 |
| predicted protein | 862 | 63 kDa | 8 |
| molybdopterin biosynthesis protein MoeA | 896 | 46 kDa | 6 |
| ferrous iron transporter A | 905 | 8 kDa | 9 |
| RadA DNA repair protein | 965 | 49 kDa | 6 |
| transcription regulator protein | 1012 | 26 kDa | 6 |
| multidrug resistance transmembrane protein | 1013 | 35 kDa | 7 |
| conserved hypothetical protein | 1156 | 25 kDa | 7 |
| conserved hypothetical protein | 1274 | 47 kDa | 6 |
| pyridoxine 5-phosphate synthase | 1331 | 28 kDa | 7 |
| ABC transporter periplasmic substrate-binding protein | 1399 | 20 kDa | 6 |
| Mg-protoporphyrin IX monomethyl ester oxidative cyclase | 1564 | 58 kDa | 9 |
| DNA polymerase III chi subunit | 1615 | 16 kDa | 6 |
| transcriptional regulator | 1738 | 34 kDa | 7 |
| conserved hypothetical protein | 1846 | 20 kDa | 7 |
| 4-diphosphocytidyl-2-C-methyl-D-erythritol kinase | 1938 | 32 kDa | 7 |
| ubiquinone/menaquinone biosynthesis methyltransferase | 1948 | 27 kDa | 7 |
| DNA polymerase III subunit delta | 1971 | 38 kDa | 9 |
| NAD-dependent protein deacetylase | 65 | 31 kDa | 5 |
| carboxymuconolactone decarboxylase | 459 | 21 kDa | 5 |
| bacteriophge tail fiber protein | 659 | 50 kDa | 6 |
| ribosomal large subunit pseudouridine synthase D | 1443 | 38 kDa | 6 |
| type III pantothenate kinase | 1832 | 28 kDa | 5 |
| ParA protein | 1805 | 28 kDa | 5 |
| conserved hypothetical protein | 1045 | 24 kDa | 3 |
| conserved hypothetical protein | 1 | 29 kDa | 5 |
| 50S ribosomal protein L29 | 2004 | 7 kDa | 4 |
| molybdenum cofactor biosynthesis protein C | 1259 | 18 kDa | 4 |
| molybdopterin-guanine dinucleotide biosynthesis protein | 51 | 19 kDa | 4 |
| transcriptional regulator | 850 | 35 kDa | 4 |
| flavin reductase domain-containing protein FMN-binding | 855 | 21 kDa | 4 |
| histone deacetylase superfamily protein | 1321 | 34 kDa | 4 |
| ribose-phosphate pyrophosphokinase | 1937 | 35 kDa | 3 |
| predicted protein | 1323 | 11 kDa | 3 |
| RNA methyltransferase | 116 | 27 kDa | 6 |
| TPR repeat-containing protein | 166 | 33 kDa | 8 |
| external-DNA catabolic protein | 171 | 33 kDa | 7 |
| methionyl aminopeptidase | 209 | 30 kDa | 7 |
| undecaprenyl pyrophosphate synthetase | 270 | 28 kDa | 7 |
| predicted protein | 362 | 28 kDa | 6 |
| outer membrane porin protein | 394 | 38 kDa | 7 |
| SAM-dependent methyltransferase | 425 | 27 kDa | 7 |
| conserved hypothetical protein | 448 | 29 kDa | 5 |
| Sel1 repeat-containing protein | 585 | 29 kDa | 6 |
| abi family protein | 600 | 37 kDa | 7 |
| Sel1 repeat-containing protein | 639 | 44 kDa | 6 |
| Sel1 repeat-containing protein | 679 | 34 kDa | 8 |
| Sel1 repeat-containing protein | 681 | 36 kDa | 9 |
| conserved hypothetical protein | 693 | 58 kDa | 9 |
| major phosphate-irrepressible acid phosphatase | 751 | 29 kDa | 6 |
| squalene/phytoene synthase | 793 | 33 kDa | 7 |
| diguanylate cyclase | 837 | 51 kDa | 7 |
| molybdopterin-guanine dinucleotide biosynthesis protein A | 895 | 25 kDa | 6 |
| nodulation protein L | 964 | 20 kDa | 6 |
| fusaric acid resistance protein region | 1015 | 76 kDa | 9 |
| esterase | 1034 | 26 kDa | 6 |
| phosphoribosylglycinamide formyltransferase | 1043 | 24 kDa | 6 |
| conserved hypothetical protein | 1072 | 37 kDa | 8 |
| ribonuclease PH/Ham1 protein | 1081 | 21 kDa | 8 |
| outer membrane autotransporter barrel domain-containing protein | 1153 | 146 kDa | 9 |
| two-component sensor kinase EnvZ | 1186 | 51 kDa | 6 |
| oxidative stress resistance two-components response regulator | 1187 | 28 kDa | 7 |
| phosphatase | 1242 | 21 kDa | 6 |
| phosphatidylserine decarboxylase | 1249 | 50 kDa | 8 |
| holo-[acyl-carrier-protein] synthase | 1330 | 16 kDa | 8 |
| tyrosine recombinase XerD | 1504 | 36 kDa | 7 |
| conserved hypothetical protein | 1545 | 40 kDa | 8 |
| exodeoxyribonuclease III | 1689 | 30 kDa | 6 |
| surface antigen | 1711 | 66 kDa | 7 |
| cyclic nucleotide-binding domain-containing protein | 1730 | 23 kDa | 8 |
| CBS domain-containing protein | 1743 | 48 kDa | 8 |
| conserved hypothetical protein | 1906 | 30 kDa | 5 |
| conserved hypothetical protein | 616 | 28 kDa | 4 |
| predicted protein | 676 | 18 kDa | 7 |
| conserved hypothetical protein | 817 | 25 kDa | 5 |
| ferrous iron transporter A | 906 | 8 kDa | 5 |
| conserved hypothetical protein | 1025 | 26 kDa | 6 |
| electron transfer flavoprotein alpha subunit | 1181 | 32 kDa | 5 |
| phosphomethylpyrimidine kinase | 1634 | 29 kDa | 6 |
| glycosyl transferase | 1747 | 31 kDa | 5 |
| tetraacyldisaccharide 4'-kinase | 2040 | 39 kDa | 5 |
| stringent starvation protein B | 1880 | 15 kDa | 5 |
| fe-s oxidoreductase | 1441 | 52 kDa | 5 |
| transcriptional regulator | 155 | 16 kDa | 5 |
| methionine biosynthesis protein MetW | 1692 | 23 kDa | 5 |
| conserved hypothetical protein | 1276 | 11 kDa | 4 |
| predicted protein | 417 | 7 kDa | 4 |
| predicted protein | 339 | 18 kDa | 3 |
| formamidase | 214 | 45 kDa | 4 |
| antibiotic biosynthesis monooxygenase | 1464 | 12 kDa | 2 |
| pyrophosphate-dependent phosphofructokinase | 3 | 46 kDa | 7 |
| conserved hypothetical protein | 16 | 35 kDa | 7 |
| conserved hypothetical protein | 91 | 40 kDa | 6 |
| glycosyl transferase family 2 protein | 223 | 35 kDa | 6 |
| conserved hypothetical protein | 228 | 23 kDa | 7 |
| condensin subunit ScpB | 255 | 21 kDa | 7 |
| predicted protein | 374 | 61 kDa | 6 |
| conserved hypothetical protein | 494 | 14 kDa | 7 |
| predicted protein | 521 | 50 kDa | 7 |
| methyltransferase | 568 | 49 kDa | 6 |
| BNR repeat containing protein | 642 | 46 kDa | 7 |
| conserved hypothetical protein | 665 | 53 kDa | 6 |
| CMP/dCMP deaminase | 924 | 18 kDa | 6 |
| ABC transporter permease | 1052 | 67 kDa | 6 |
| DNA-binding response regulator | 1056 | 27 kDa | 6 |
| UDP-glucose 4-epimerase | 1088 | 37 kDa | 6 |
| conserved hypothetical protein | 1124 | 22 kDa | 6 |
| predicted protein | 1178 | 22 kDa | 6 |
| predicted protein | 1244 | 33 kDa | 6 |
| conserved hypothetical protein | 1273 | 35 kDa | 7 |
| conserved hypothetical protein | 1349 | 39 kDa | 7 |
| Sel1 repeat-containing protein | 1374 | 26 kDa | 6 |
| outer membrane autotransporter barrel domain-containing protein | 1404 | 90 kDa | 8 |
| adenine phosphoribosyltransferase | 1671 | 19 kDa | 6 |
| sulfatase | 1777 | 56 kDa | 6 |
| leader peptidase | 1788 | 33 kDa | 6 |
| phosphoglycolate phosphatase | 1790 | 29 kDa | 7 |
| phosphoribosyl-AMP cyclohydrolase | 1886 | 15 kDa | 7 |
| TPR repeat-containing protein | 1920 | 35 kDa | 7 |
| Na+/solute symporter | 1951 | 53 kDa | 6 |
| biotin synthesis protein bioC | 2056 | 32 kDa | 6 |
| methylase | 2063 | 25 kDa | 5 |
| glucokinase | 234 | 36 kDa | 5 |
| helix-turn-helix domain-containing protein | 1432 | 14 kDa | 6 |
| predicted protein | 754 | 52 kDa | 5 |
| HIT family hydrolase | 1947 | 17 kDa | 5 |
| response regulator receiver protein | 141 | 22 kDa | 4 |
| uroporphyrin-III C/tetrapyrrole methyltransferase | 172 | 27 kDa | 5 |
| thioesterase | 1284 | 16 kDa | 5 |
| A/G-specific adenine glycosylase | 1940 | 42 kDa | 5 |
| dTDP-4-dehydrorhamnose 3,5-epimerase | 1077 | 22 kDa | 4 |
| predicted protein | 916 | 13 kDa | 5 |
| arginase | 456 | 33 kDa | 5 |
| conserved hypothetical protein | 997 | 35 kDa | 4 |
| transcriptional regulator | 37 | 17 kDa | 4 |
| conserved hypothetical protein | 1864 | 20 kDa | 5 |
| sodium/proton antiporter | 1253 | 66 kDa | 4 |
| conserved hypothetical protein | 1572 | 24 kDa | 4 |
| conserved hypothetical protein | 1696 | 14 kDa | 4 |
| site specific integrase/recombinase | 1295 | 36 kDa | 3 |
| Fe | 96 | 11 kDa | 3 |
| ribonuclease H | 1210 | 16 kDa | 4 |
| methylated-DNA-protein-cysteine methyltransferase | 824 | 18 kDa | 3 |
| ABC-2 type transporter | 1148 | 42 kDa | 4 |
| 50S ribosomal subunit protein L34 | 1794 | 6 kDa | 4 |
| predicted protein | 5 | 11 kDa | 3 |
| RNA-binding protein | 195 | 9 kDa | 3 |
| predicted protein | 305 | 27 kDa | 3 |
| peptidylprolyl isomerase | 644 | 18 kDa | 3 |
| cell division protein | 1659 | 9 kDa | 2 |
| phosphoserine aminotransferase | 76 | 41 kDa | 8 |
| RNA polymerase sigma-E factor | 182 | 23 kDa | 6 |
| phage major capsid protein | 323 | 44 kDa | 6 |
| conserved hypothetical protein | 392 | 16 kDa | 6 |
| conserved hypothetical protein | 534 | 23 kDa | 9 |
| predicted protein | 627 | 45 kDa | 7 |
| conserved hypothetical protein | 684 | 15 kDa | 7 |
| transcriptional regulator | 759 | 13 kDa | 6 |
| conserved hypothetical protein | 797 | 28 kDa | 5 |
| PhyA2 protein | 852 | 45 kDa | 7 |
| predicted protein | 917 | 34 kDa | 7 |
| Sel1 domain-containing protein | 936 | 45 kDa | 7 |
| outer membrane autotransporter barrel domain-containing protein | 968 | 59 kDa | 7 |
| methyltransferase HemK MTase hemK | 1053 | 30 kDa | 7 |
| ribose-5-phosphate isomerase A | 1254 | 24 kDa | 7 |
| segregation and condensation protein A | 1267 | 32 kDa | 6 |
| outer membrane porin protein | 1297 | 41 kDa | 7 |
| NADH dehydrogenase chain F | 1367 | 47 kDa | 9 |
| acetate kinase | 1476 | 43 kDa | 7 |
| octaprenyl-diphosphate synthase | 1742 | 36 kDa | 6 |
| predicted protein | 1835 | 32 kDa | 8 |
| predicted protein | 2066 | 44 kDa | 7 |
| 1-deoxy-D-xylulose-5-phosphate reductoisomerase | 272 | 43 kDa | 6 |
| tail fiber protein gpH | 348 | 42 kDa | 6 |
| conserved hypothetical protein | 398 | 24 kDa | 6 |
| BNR repeat containing protein | 621 | 44 kDa | 6 |
| 4-hydroxythreonine-4-phosphate dehydrogenase | 1548 | 38 kDa | 5 |
| shikimate dehydrogenase | 1567 | 29 kDa | 6 |
| phosphonate dehydrogenase | 1761 | 37 kDa | 6 |
| ABC transport system permease | 1901 | 28 kDa | 6 |
| electron transport complex protein | 258 | 25 kDa | 5 |
| cardiolipin synthetase II | 1660 | 45 kDa | 4 |
| conserved hypothetical protein | 26 | 23 kDa | 4 |
| minor tail protein L | 707 | 26 kDa | 5 |
| predicted protein | 1452 | 13 kDa | 5 |
| ribulose-phosphate 3-epimerase | 1591 | 25 kDa | 5 |
| queuine/archaeosine tRNA-ribosyltransferase | 1852 | 42 kDa | 5 |
| conserved hypothetical protein | 1411 | 41 kDa | 4 |
| predicted protein | 366 | 20 kDa | 5 |
| predicted protein | 920 | 18 kDa | 5 |
| lipoprotein releasing system ATP-binding protein lolD | 932 | 25 kDa | 5 |
| phospholipase D/transphosphatidylase | 1632 | 55 kDa | 5 |
| F0F1 ATP synthase subunit A | 1829 | 31 kDa | 5 |
| FdsC protein | 60 | 29 kDa | 4 |
| predicted protein | 1130 | 23 kDa | 5 |
| tetraacyldisaccharide-1-P synthase | 279 | 41 kDa | 5 |
| Sel1 repeat-containing protein | 502 | 34 kDa | 4 |
| predicted protein | 211 | 21 kDa | 3 |
| transcriptional regulator | 830 | 34 kDa | 4 |
| conserved hypothetical protein | 1538 | 20 kDa | 4 |
| competence/damage inducible protein CinA C-terminal domain | 1560 | 18 kDa | 4 |
| phosphotransferase domain-containing protein | 1173 | 30 kDa | 4 |
| ferredoxin protein | 31 | 12 kDa | 3 |
| HAD-superfamily subfamily IB hydrolase | 1515 | 25 kDa | 3 |
| bacteriophage lambda tail assembly protein I | 709 | 21 kDa | 4 |
| predicted protein | 114 | 27 kDa | 3 |
| short-chain dehydrogenase/reductase SDR | 833 | 26 kDa | 2 |
| predicted protein | 1252 | 17 kDa | 3 |
| endoribonuclease L-PSP | 1232 | 16 kDa | 2 |
| predicted protein | 1403 | 13 kDa | 2 |
| lipopolysaccharide 3-alpha-galactosyltransferase | 226 | 36 kDa | 7 |
| conserved hypothetical protein | 335 | 96 kDa | 7 |
| aldo/keto reductase | 428 | 36 kDa | 6 |
| predicted protein | 482 | 34 kDa | 7 |
| conserved hypothetical protein | 500 | 36 kDa | 6 |
| predicted protein | 506 | 14 kDa | 6 |
| predicted protein | 589 | 32 kDa | 6 |
| bacteriophge tail fiber protein | 623 | 42 kDa | 6 |
| conserved hypothetical protein | 783 | 32 kDa | 6 |
| predicted protein | 857 | 19 kDa | 6 |
| outer membrane lipoproteins ABC transporter membrane protein | 933 | 46 kDa | 5 |
| aminodeoxychorismate lyase | 943 | 36 kDa | 6 |
| conserved hypothetical protein | 961 | 49 kDa | 6 |
| transferase hexapeptide repeat-containing protein | 1175 | 21 kDa | 6 |
| translation factor SUA5 | 1196 | 37 kDa | 8 |
| 1-acyl-sn-glycerol-3-phosphate acyltransferase | 1243 | 28 kDa | 7 |
| kef-type K+ transport system | 1395 | 73 kDa | 6 |
| transcriptional regulator | 594 | 16 kDa | 5 |
| conserved hypothetical protein | 608 | 28 kDa | 4 |
| macrolide-specific efflux protein MacA | 13 | 44 kDa | 5 |
| HhH-GPD family protein | 124 | 24 kDa | 5 |
| phage tail collar protein | 656 | 39 kDa | 5 |
| DNA repair protein recO | 1332 | 30 kDa | 5 |
| conserved hypothetical protein | 668 | 23 kDa | 4 |
| 3-methyl-2-oxobutanoate hydroxymethyltransferase | 1519 | 29 kDa | 5 |
| acyl transferase domain-containing protein | 829 | 33 kDa | 6 |
| Cd | 959 | 19 kDa | 5 |
| chloramphenicol acetyltransferase | 696 | 24 kDa | 5 |
| conserved hypothetical protein | 1092 | 45 kDa | 5 |
| ferric uptake regulator protein | 1976 | 16 kDa | 5 |
| glycine cleavage T-protein superfamily protein | 944 | 36 kDa | 5 |
| conserved hypothetical protein | 1117 | 18 kDa | 4 |
| carboxymuconolactone decarboxylase | 847 | 26 kDa | 5 |
| N utilization substance protein B | 1725 | 18 kDa | 5 |
| predicted protein | 712 | 41 kDa | 5 |
| conserved hypothetical protein | 611 | 11 kDa | 5 |
| peptidase family M48 protein | 1665 | 47 kDa | 4 |
| D-isomer specific 2-hydroxyacid dehydrogenase NAD-binding | 1622 | 35 kDa | 4 |
| conserved hypothetical protein | 332 | 15 kDa | 4 |
| endonuclease Nuc | 805 | 20 kDa | 4 |
| NUDIX hydrolase | 1470 | 26 kDa | 4 |
| conserved hypothetical protein | 53 | 15 kDa | 4 |
| ABC-type multidrug transport system | 1896 | 34 kDa | 4 |
| serine protease | 185 | 41 kDa | 3 |
| aldo/keto reductase | 866 | 36 kDa | 2 |
| transcriptional regulator | 315 | 13 kDa | 3 |
| conserved hypothetical protein | 1882 | 29 kDa | 3 |
| tRNA pseudouridine synthase B | 1223 | 35 kDa | 2 |
| ribonucleoside-triphosphate reductase | 1744 | 26 kDa | 3 |
| predicted protein | 892 | 20 kDa | 2 |
| sulphate transporter | 1388 | 62 kDa | 2 |
| radical SAM domain-containing protein | 445 | 31 kDa | 6 |
| outer membrane porin protein | 576 | 39 kDa | 6 |
| AraC family transcriptional regulator | 740 | 33 kDa | 6 |
| predicted protein | 818 | 59 kDa | 8 |
| 3-methyladenine DNA glycosylase | 973 | 22 kDa | 7 |
| conserved hypothetical protein | 988 | 76 kDa | 5 |
| outer membrane porin protein | 1437 | 37 kDa | 6 |
| transglycosylase | 1585 | 33 kDa | 6 |
| conserved hypothetical protein | 1716 | 28 kDa | 6 |
| hpr serine kinase/phosphatase | 1942 | 35 kDa | 6 |
| DUF488 domain-containing protein | 543 | 14 kDa | 5 |
| transcriptional regulator | 695 | 33 kDa | 6 |
| conserved hypothetical protein | 986 | 77 kDa | 6 |
| 2,3-diketo-5-methylthio-1-phosphopentane phosphatase | 1405 | 26 kDa | 6 |
| Sel1 repeat-containing protein | 467 | 37 kDa | 4 |
| ATP-dependent DNA ligase | 800 | 36 kDa | 5 |
| RND efflux system outer membrane lipoprotein | 948 | 53 kDa | 5 |
| conserved hypothetical protein | 1089 | 33 kDa | 6 |
| methionyl-tRNA formyltransferase | 1729 | 34 kDa | 5 |
| 3-octaprenyl-4-hydroxybenzoate carboxy-lyase | 1868 | 21 kDa | 6 |
| oxidoreductase | 566 | 43 kDa | 5 |
| conserved hypothetical protein | 657 | 39 kDa | 5 |
| D-xylose ABC transporter | 353 | 37 kDa | 5 |
| conserved hypothetical protein | 1415 | 19 kDa | 5 |
| hydrolase | 841 | 24 kDa | 4 |
| conserved hypothetical protein | 513 | 50 kDa | 5 |
| enoyl-CoA hydratase | 162 | 31 kDa | 4 |
| outer membrane protein A | 74 | 22 kDa | 4 |
| outer membrane porin protein | 491 | 42 kDa | 5 |
| DNA internalization-related competence protein | 930 | 90 kDa | 5 |
| Sel1 repeat-containing protein | 580 | 35 kDa | 5 |
| conserved hypothetical protein | 634 | 21 kDa | 4 |
| membrane-bound lytic Murein transglycosylase B | 1719 | 42 kDa | 3 |
| diadenosine tetraphosphatase | 1293 | 31 kDa | 4 |
| conserved hypothetical protein | 1350 | 27 kDa | 5 |
| heat-inducible transcriptional repressor | 2048 | 37 kDa | 4 |
| thiol:disulfide interchange protein DsbC | 1154 | 26 kDa | 4 |
| predicted protein | 1587 | 11 kDa | 4 |
| dithiobiotin synthetase | 1620 | 24 kDa | 4 |
| rare lipoprotein A | 1301 | 36 kDa | 4 |
| tmRNA-binding protein small potein B | 29 | 17 kDa | 5 |
| heat shock protein | 100 | 15 kDa | 4 |
| conserved hypothetical protein | 11 | 51 kDa | 3 |
| organic radical activating enzyme | 1118 | 24 kDa | 4 |
| carboxylesterase bioH | 951 | 27 kDa | 5 |
| conserved hypothetical protein | 1138 | 39 kDa | 3 |
| conserved hypothetical protein | 349 | 24 kDa | 4 |
| ferredoxin oxidoreductase | 1766 | 39 kDa | 3 |
| beta-lactamase | 689 | 34 kDa | 4 |
| L-isoaspartate O-methyltransferase | 157 | 31 kDa | 4 |
| predicted protein | 1863 | 11 kDa | 4 |
| predicted protein | 629 | 14 kDa | 4 |
| methyltransferase | 1475 | 29 kDa | 4 |
| conserved hypothetical protein | 517 | 16 kDa | 3 |
| Mg-dependent DNase | 931 | 30 kDa | 3 |
| conserved hypothetical protein | 1385 | 28 kDa | 3 |
| RNA methyltransferase | 1325 | 29 kDa | 4 |
| conserved hypothetical protein | 1933 | 12 kDa | 3 |
| conserved hypothetical protein | 294 | 18 kDa | 3 |
| MarR family transcriptional regulator | 985 | 17 kDa | 4 |
| malonate decarboxylase gamma subunit | 828 | 31 kDa | 2 |
| predicted protein | 721 | 18 kDa | 3 |
| conserved hypothetical protein | 617 | 51 kDa | 3 |
| predicted protein | 691 | 15 kDa | 3 |
| membrane protein | 582 | 21 kDa | 3 |
| glycosyltransferase | 1189 | 65 kDa | 2 |
| conserved hypothetical protein | 1216 | 31 kDa | 2 |
| transporter for D-alanine | 607 | 53 kDa | 6 |
| predicted protein | 753 | 50 kDa | 6 |
| conserved hypothetical protein | 806 | 21 kDa | 6 |
| septum formation initiator | 926 | 16 kDa | 6 |
| methyltransferase type 11 | 1179 | 30 kDa | 7 |
| 2,3,4,5-tetrahydropyridine-2-carboxylate N-succinyltransferase | 1206 | 31 kDa | 6 |
| NADH-quinone oxidoreductase subunit I | 1364 | 19 kDa | 6 |
| phosphatidylserine synthase | 103 | 29 kDa | 5 |
| RmlA protein | 1078 | 35 kDa | 6 |
| DegT/DnrJ/EryC1/StrS aminotransferase | 1409 | 44 kDa | 5 |
| nitroreductase | 377 | 22 kDa | 5 |
| ABC-type phosphate transport system substrate-binding protein | 409 | 36 kDa | 5 |
| conserved hypothetical protein | 868 | 27 kDa | 5 |
| predicted protein | 1352 | 21 kDa | 6 |
| zinc-binding dehydrogenase | 360 | 36 kDa | 6 |
| predicted protein | 774 | 41 kDa | 5 |
| outer membrane porin protein | 391 | 39 kDa | 5 |
| dihydrofolate reductase | 82 | 18 kDa | 5 |
| ferredoxin | 260 | 12 kDa | 5 |
| membrane-fusion protein | 591 | 37 kDa | 5 |
| conserved hypothetical protein | 628 | 22 kDa | 4 |
| sodium/proline symporter PutP | 1491 | 57 kDa | 5 |
| heme utilization protein HuvX | 1046 | 20 kDa | 4 |
| tRNA | 1472 | 29 kDa | 5 |
| undecaprenyl-diphosphatase | 14 | 30 kDa | 5 |
| tRNA and rRNA cytosine-C5-methylase | 1594 | 49 kDa | 5 |
| conserved hypothetical protein | 346 | 69 kDa | 4 |
| predicted protein | 466 | 11 kDa | 4 |
| Sel1 repeat-containing protein | 680 | 21 kDa | 5 |
| predicted protein | 522 | 24 kDa | 4 |
| conserved hypothetical protein | 1004 | 52 kDa | 5 |
| thiamine pyrophosphate enzyme TPP binding domain-containing protein | 1480 | 59 kDa | 4 |
| ribonuclease HII | 280 | 24 kDa | 5 |
| Sel1 repeat-containing protein | 503 | 12 kDa | 5 |
| NADH-quinone oxidoreductase subunit B | 1371 | 18 kDa | 4 |
| MarR family transcriptional regulator | 987 | 16 kDa | 5 |
| conserved hypothetical protein | 1482 | 26 kDa | 4 |
| conserved hypothetical protein | 1033 | 22 kDa | 4 |
| membrane protein Psyr_1725 | 772 | 22 kDa | 4 |
| hypothetical protein | 638 | 26 kDa | 4 |
| predicted protein | 722 | 40 kDa | 4 |
| conserved hypothetical protein | 919 | 10 kDa | 5 |
| transcriptional regulator | 1277 | 30 kDa | 4 |
| conserved hypothetical protein | 1908 | 14 kDa | 4 |
| conserved hypothetical protein | 281 | 28 kDa | 5 |
| ferrous iron transporter B | 904 | 79 kDa | 4 |
| nucleotidyltransferase | 1070 | 27 kDa | 4 |
| conserved hypothetical protein | 1143 | 18 kDa | 4 |
| conserved hypothetical protein | 1026 | 48 kDa | 4 |
| conserved hypothetical protein | 1771 | 15 kDa | 4 |
| transcriptional regulator | 685 | 18 kDa | 3 |
| 5-formyltetrahydrofolate cyclo-ligase | 1912 | 22 kDa | 4 |
| conserved hypothetical protein | 1410 | 33 kDa | 4 |
| predicted protein | 1478 | 36 kDa | 4 |
| phosphonate transport system substrate-binding protein | 1763 | 31 kDa | 3 |
| recombinational DNA repair protein | 1581 | 22 kDa | 4 |
| conserved hypothetical protein | 371 | 14 kDa | 5 |
| methyltransferase type 11 | 1417 | 23 kDa | 3 |
| conserved hypothetical protein | 1219 | 16 kDa | 3 |
| plasmid maintenance system killer | 578 | 11 kDa | 3 |
| molybdopterin synthase subunit MoaD | 1125 | 10 kDa | 3 |
| HicB protein | 4 | 19 kDa | 3 |
| predicted protein | 1616 | 17 kDa | 3 |
| predicted protein | 803 | 21 kDa | 3 |
| ABC-type molybdate transport system substrate-binding protein | 58 | 27 kDa | 3 |
| translation initiation factor IF-1 | 2017 | 8 kDa | 3 |
| recombination regulator RecX | 2034 | 17 kDa | 2 |
| predicted protein | 1932 | 8 kDa | 2 |
| biopolymer transporter | 1283 | 25 kDa | 2 |
| lipoprotein | 1836 | 23 kDa | 2 |
| 4-hydroxybenzoate polyprenyl transferase | 1856 | 32 kDa | 2 |
| predicted protein | 1657 | 10 kDa | 2 |
| predicted protein | 744 | 43 kDa | 6 |
| Sel1 repeat-containing protein | 745 | 36 kDa | 6 |
| OmpA/MotB domain-containing protein | 1087 | 22 kDa | 6 |
| conserved hypothetical protein | 1568 | 30 kDa | 6 |
| conserved hypothetical protein | 1618 | 15 kDa | 5 |
| predicted protein | 1967 | 30 kDa | 6 |
| predicted protein | 705 | 39 kDa | 6 |
| NADH dehydrogenase subunit I E | 1368 | 17 kDa | 5 |
| NLP/P60 family protein | 242 | 23 kDa | 5 |
| predicted protein | 86 | 15 kDa | 5 |
| conserved hypothetical protein | 174 | 18 kDa | 5 |
| conserved hypothetical protein | 1007 | 20 kDa | 6 |
| 4-oxalocrotonate tautomerase | 1756 | 7 kDa | 5 |
| predicted protein | 145 | 74 kDa | 4 |
| small heat shock protein | 1386 | 17 kDa | 5 |
| conserved hypothetical protein | 105 | 19 kDa | 5 |
| outer membrane autotransporter barrel domain-containing protein | 1218 | 67 kDa | 4 |
| predicted protein | 1775 | 23 kDa | 5 |
| conserved hypothetical protein | 306 | 25 kDa | 4 |
| conserved hypothetical protein | 1074 | 16 kDa | 4 |
| conserved hypothetical protein | 1709 | 27 kDa | 4 |
| conserved hypothetical protein | 1770 | 32 kDa | 5 |
| ferrous iron transporter B:Small GTP-binding protein domain | 1981 | 68 kDa | 4 |
| UTP-glucose-1-phosphate uridylyltransferase | 1237 | 36 kDa | 4 |
| ADP-ribose pyrophosphatase | 1493 | 21 kDa | 4 |
| conserved hypothetical protein | 313 | 31 kDa | 4 |
| ribosomal RNA large subunit methyltransferase J | 1164 | 24 kDa | 4 |
| conserved hypothetical protein | 334 | 15 kDa | 4 |
| endonuclease III | 257 | 24 kDa | 4 |
| NADPH-dependent fmn reductase | 1150 | 21 kDa | 4 |
| glutathione S-transferase domain-containing protein | 1498 | 22 kDa | 4 |
| conserved hypothetical protein | 949 | 28 kDa | 4 |
| conserved hypothetical protein | 296 | 12 kDa | 4 |
| predicted protein | 567 | 49 kDa | 3 |
| conserved hypothetical protein | 667 | 33 kDa | 4 |
| conserved hypothetical protein | 1785 | 7 kDa | 3 |
| surface lipoprotein | 1899 | 36 kDa | 3 |
| rhodanese domain-containing protein | 962 | 19 kDa | 3 |
| predicted protein | 1818 | 15 kDa | 3 |
| transcriptional regulator | 1458 | 9 kDa | 3 |
| toluene efflux pump periplasmic linker protein ttgA | 946 | 44 kDa | 3 |
| bacteriophage protein | 1005 | 21 kDa | 4 |
| predicted protein | 782 | 43 kDa | 2 |
| conserved hypothetical protein | 312 | 17 kDa | 3 |
| conserved hypothetical protein | 361 | 14 kDa | 3 |
| conserved hypothetical protein | 702 | 14 kDa | 3 |
| predicted protein | 775 | 58 kDa | 3 |
| ADP-heptose synthase | 1707 | 17 kDa | 3 |
| bacteriophge tail fiber protein | 918 | 50 kDa | 3 |
| ferredoxin | 20 | 13 kDa | 3 |
| conserved hypothetical protein | 449 | 29 kDa | 2 |
| conserved hypothetical protein | 284 | 16 kDa | 3 |
| conserved hypothetical protein | 718 | 21 kDa | 3 |
| conserved hypothetical protein | 981 | 17 kDa | 3 |
| conserved hypothetical protein | 860 | 11 kDa | 2 |
| amidophosphoribosyltransferase | 2055 | 29 kDa | 2 |
| predicted protein | 1157 | 20 kDa | 2 |
| conserved hypothetical protein | 1640 | 33 kDa | 3 |
| predicted protein | 1551 | 13 kDa | 2 |
| conserved hypothetical protein | 1869 | 40 kDa | 2 |
| TPR repeat-containing protein | 253 | 34 kDa | 5 |
| two component transcriptional regulator | 1873 | 26 kDa | 5 |
| peptide methionine sulfoxide reductase | 1194 | 20 kDa | 5 |
| tRNA 2-thiocytidine biosynthesis protein ttcA | 1706 | 35 kDa | 5 |
| predicted protein | 329 | 11 kDa | 6 |
| glycosyltransferase tibC | 854 | 48 kDa | 4 |
| MarR family transcriptional regulator | 1978 | 19 kDa | 5 |
| conserved hypothetical protein | 675 | 20 kDa | 5 |
| BNR repeat containing protein | 633 | 40 kDa | 4 |
| polysaccharide deacetylase | 738 | 34 kDa | 4 |
| SPOUT methyltransferase superfamily protein | 229 | 18 kDa | 4 |
| predicted protein | 462 | 22 kDa | 4 |
| two-component system sensory histidine kinase | 240 | 40 kDa | 4 |
| N-acetyl-anhydromuranmyl-L-alanine amidase | 1862 | 22 kDa | 4 |
| intracellular septation protein A | 161 | 24 kDa | 4 |
| predicted protein | 225 | 49 kDa | 3 |
| gp64 | 708 | 27 kDa | 4 |
| conserved hypothetical protein | 531 | 10 kDa | 4 |
| GTP-binding protein | 1717 | 33 kDa | 3 |
| conserved hypothetical protein | 1017 | 21 kDa | 4 |
| conserved hypothetical protein | 2032 | 22 kDa | 4 |
| predicted protein | 662 | 20 kDa | 4 |
| conserved hypothetical protein | 309 | 18 kDa | 4 |
| conserved hypothetical protein | 730 | 11 kDa | 3 |
| conserved hypothetical protein | 658 | 12 kDa | 2 |
| peptide methionine sulfoxide reductase | 160 | 16 kDa | 3 |
| plasmid maintenance system antidote protein | 577 | 11 kDa | 4 |
| DNA repair protein radC | 236 | 25 kDa | 3 |
| acriflavin resistance periplasmic protein | 473 | 47 kDa | 3 |
| MdcC protein | 827 | 11 kDa | 4 |
| small conductance mechanosensitive ion channel family protein | 210 | 39 kDa | 3 |
| conserved hypothetical protein | 963 | 19 kDa | 2 |
| predicted protein | 739 | 23 kDa | 3 |
| conserved hypothetical protein | 1962 | 21 kDa | 3 |
| predicted protein | 723 | 23 kDa | 3 |
| predicted protein | 1859 | 12 kDa | 3 |
| maf protein | 173 | 22 kDa | 3 |
| NAD-dependent epimerase/dehydratase | 478 | 24 kDa | 3 |
| conserved hypothetical protein | 1699 | 10 kDa | 3 |
| conserved hypothetical protein | 25 | 15 kDa | 3 |
| predicted protein | 469 | 26 kDa | 3 |
| SAM-dependent methyltransferase | 601 | 27 kDa | 3 |
| conserved hypothetical protein | 760 | 15 kDa | 3 |
| bifunctional enzyme ispD/ispF | 1136 | 27 kDa | 3 |
| transcriptional regulator | 1922 | 10 kDa | 3 |
| PTS system nitrogen regulatory IIA protein | 1943 | 17 kDa | 3 |
| predicted protein | 1982 | 11 kDa | 3 |
| conserved hypothetical protein | 996 | 25 kDa | 2 |
| drug resistance transporter | 1014 | 57 kDa | 3 |
| conserved hypothetical protein | 773 | 47 kDa | 2 |
| predicted protein | 490 | 9 kDa | 2 |
| acyltransferase | 654 | 19 kDa | 2 |
| conserved hypothetical protein | 395 | 29 kDa | 2 |
| transcriptional regulator | 528 | 16 kDa | 3 |
| ribonuclease BN transmembrane protein | 954 | 49 kDa | 2 |
| acetyltransferase | 620 | 18 kDa | 2 |
| predicted protein | 625 | 10 kDa | 2 |
| tRNA | 110 | 28 kDa | 2 |
| conserved hypothetical protein | 92 | 43 kDa | 2 |
| predicted protein | 352 | 51 kDa | 2 |
| conserved hypothetical protein | 2054 | 18 kDa | 2 |
| conserved hypothetical protein | 573 | 14 kDa | 2 |
| FKBP-type peptidyl-prolyl cis-trans isomerase | 1378 | 19 kDa | 2 |
| TOBE domain-containing protein | 1520 | 7 kDa | 2 |
| phosphoribosylanthranilate isomerase | 1346 | 24 kDa | 5 |
| potassium transport system | 1904 | 69 kDa | 5 |
| tRNA-specific adenosine deaminase | 1116 | 19 kDa | 4 |
| glycoside hydrolase | 338 | 18 kDa | 4 |
| conserved hypothetical protein | 1522 | 33 kDa | 5 |
| outer membrane protein | 9 | 48 kDa | 4 |
| peptide deformylase | 206 | 20 kDa | 4 |
| G:T/U mismatch-specific DNA glycosylase | 789 | 18 kDa | 4 |
| predicted protein | 319 | 12 kDa | 4 |
| predicted protein | 418 | 28 kDa | 4 |
| conserved hypothetical protein | 1151 | 11 kDa | 4 |
| conserved hypothetical protein | 216 | 34 kDa | 4 |
| predicted protein | 1047 | 30 kDa | 4 |
| conserved hypothetical protein | 666 | 16 kDa | 4 |
| apolipoprotein N-acyltransferase | 612 | 56 kDa | 4 |
| phage tail collar domain-containing protein | 716 | 46 kDa | 3 |
| conserved hypothetical protein | 1798 | 42 kDa | 3 |
| conserved hypothetical protein | 1643 | 17 kDa | 3 |
| proton-translocating NADH-quinone oxidoreductase | 1361 | 76 kDa | 3 |
| phosphonates import ATP-binding protein PhnC | 1764 | 29 kDa | 4 |
| prolipoprotein diacylglyceryl transferase | 2035 | 31 kDa | 4 |
| conserved hypothetical protein | 376 | 28 kDa | 3 |
| Sel1 repeat-containing protein | 682 | 18 kDa | 3 |
| PRC-barrel domain-containing protein | 84 | 15 kDa | 3 |
| multi antimicrobial extrusion protein MatE | 549 | 50 kDa | 3 |
| L-isoaspartate O-methyltransferase | 10 | 25 kDa | 4 |
| conserved hypothetical protein | 1570 | 22 kDa | 3 |
| single-strand binding protein | 802 | 16 kDa | 4 |
| predicted protein | 563 | 11 kDa | 2 |
| predicted protein | 717 | 12 kDa | 3 |
| predicted protein | 436 | 9 kDa | 3 |
| methyltransferase | 873 | 24 kDa | 3 |
| magnesium and cobalt transporter CorA | 438 | 44 kDa | 3 |
| predicted protein | 714 | 57 kDa | 3 |
| predicted protein | 715 | 10 kDa | 3 |
| lipopolysaccharide core biosynthesis glycosyltransferase | 219 | 12 kDa | 3 |
| predicted protein | 297 | 16 kDa | 3 |
| transcriptional regulator | 440 | 14 kDa | 3 |
| predicted protein | 441 | 11 kDa | 3 |
| predicted protein | 663 | 7 kDa | 3 |
| predicted protein | 861 | 7 kDa | 3 |
| conserved hypothetical protein | 1456 | 25 kDa | 3 |
| ribosome maturation factor rimM | 1473 | 20 kDa | 3 |
| MutT/NUDIX family hydrolase | 1542 | 18 kDa | 3 |
| predicted protein | 1059 | 17 kDa | 3 |
| guanylate kinase | 1445 | 24 kDa | 3 |
| predicted protein | 840 | 12 kDa | 3 |
| predicted protein | 1233 | 15 kDa | 3 |
| conserved hypothetical protein | 1326 | 21 kDa | 3 |
| conserved hypothetical protein | 843 | 10 kDa | 3 |
| predicted protein | 439 | 15 kDa | 2 |
| dihydroneopterin aldolase | 1705 | 15 kDa | 3 |
| DUF185 domain-containing protein | 1703 | 42 kDa | 3 |
| protein-tyrosine-phosphatase | 779 | 17 kDa | 3 |
| MltA-interacting protein | 1413 | 28 kDa | 2 |
| anthranilate synthase component II | 1784 | 21 kDa | 2 |
| multi antimicrobial extrusion protein MatE | 550 | 52 kDa | 2 |
| conserved hypothetical protein | 1941 | 33 kDa | 2 |
| rod shape-determining protein RodA | 1302 | 41 kDa | 2 |
| predicted protein | 511 | 20 kDa | 3 |
| Na+/H+ antiporter | 1757 | 46 kDa | 2 |
| bacteriophge tail fiber protein | 766 | 41 kDa | 2 |
| major Facilitator Superfamily protein | 435 | 41 kDa | 2 |
| alpha amylase domain-containing protein | 443 | 20 kDa | 2 |
| predicted protein | 1058 | 18 kDa | 2 |
| predicted protein | 1122 | 9 kDa | 2 |
| phospho-N-acetylmuramoyl-pentapeptide-transferase | 1653 | 43 kDa | 2 |
| conserved hypothetical protein | 2041 | 7 kDa | 2 |
| periplasmic divalent cation tolerance protein | 2023 | 12 kDa | 2 |
| predicted protein | 368 | 12 kDa | 2 |
| RuvA protein | 1461 | 21 kDa | 2 |
| conserved hypothetical protein | 298 | 19 kDa | 4 |
| L-asparaginase | 1715 | 18 kDa | 4 |
| predicted protein | 518 | 19 kDa | 4 |
| predicted protein | 1466 | 48 kDa | 4 |
| phosphate transport system permease PstC | 410 | 37 kDa | 4 |
| carbon starvation protein A | 640 | 52 kDa | 4 |
| predicted protein | 317 | 20 kDa | 3 |
| 1-acyl-sn-glycerol-3-phosphate acyltransferase | 1292 | 29 kDa | 3 |
| ribulose-phosphate 3-epimerase | 1068 | 25 kDa | 3 |
| conserved hypothetical protein | 1392 | 46 kDa | 3 |
| biopolymer transporter TolR | 1282 | 16 kDa | 3 |
| autotransporter | 474 | 52 kDa | 4 |
| predicted protein | 661 | 50 kDa | 3 |
| conserved hypothetical protein | 1285 | 27 kDa | 4 |
| predicted protein | 437 | 18 kDa | 3 |
| conserved hypothetical protein | 1348 | 16 kDa | 3 |
| conserved hypothetical protein | 397 | 36 kDa | 3 |
| predicted protein | 390 | 39 kDa | 3 |
| GreA/GreB family elongation factor | 839 | 15 kDa | 3 |
| TriP hydrolase domain-containing protein | 1507 | 18 kDa | 3 |
| 4Fe-4S ferredoxin iron-sulfur binding domain-containing protein | 2065 | 9 kDa | 3 |
| helix-turn-helix domain-containing protein | 1020 | 34 kDa | 3 |
| conserved hypothetical protein | 168 | 18 kDa | 3 |
| conserved hypothetical protein | 994 | 41 kDa | 3 |
| predicted protein | 992 | 49 kDa | 3 |
| predicted protein | 442 | 19 kDa | 3 |
| BNR repeat containing protein | 645 | 42 kDa | 3 |
| predicted protein | 1488 | 54 kDa | 3 |
| 2',5' RNA ligase | 1191 | 24 kDa | 2 |
| predicted protein | 546 | 15 kDa | 2 |
| oligopeptide transport system permease appC | 1959 | 41 kDa | 3 |
| BNR repeat containing protein | 641 | 45 kDa | 2 |
| drug resistance transporter EmrB/QacA subfamily protein | 752 | 52 kDa | 2 |
| ribosomal RNA small subunit methyltransferase G | 1806 | 25 kDa | 3 |
| octanoyltransferase | 1840 | 26 kDa | 3 |
| thioesterase | 1732 | 15 kDa | 3 |
| predicted protein | 1418 | 7 kDa | 3 |
| tRNA/rRNA methyltransferase | 1094 | 29 kDa | 3 |
| type 1 pili usher pathway chaperone CsuC | 910 | 29 kDa | 3 |
| transcriptional regulator | 1373 | 12 kDa | 2 |
| predicted protein | 80 | 9 kDa | 3 |
| crossover junction endodeoxyribonuclease ruvC | 1460 | 19 kDa | 3 |
| mutator MutT protein | 1463 | 17 kDa | 3 |
| FtsW cell division protein | 1651 | 45 kDa | 3 |
| predicted protein | 713 | 57 kDa | 2 |
| alpha/beta hydrolase fold-containing protein | 1406 | 32 kDa | 2 |
| sulfate adenylyltransferase | 146 | 26 kDa | 2 |
| predicted protein | 520 | 10 kDa | 2 |
| ABC transport system permease | 1833 | 41 kDa | 2 |
| conserved hypothetical protein | 995 | 13 kDa | 2 |
| conserved hypothetical protein | 314 | 11 kDa | 2 |
| S-CspCI protein | 7 | 35 kDa | 2 |
| outer membrane autotransporter barrel domain-containing protein | 823 | 64 kDa | 2 |
| conserved hypothetical protein | 112 | 10 kDa | 2 |
| modification methylase HemK | 1208 | 34 kDa | 3 |
| helix-turn-helix domain-containing protein | 342 | 12 kDa | 2 |
| NADH-ubiquinone oxidoreductase | 1359 | 54 kDa | 2 |
| conserved hypothetical protein | 880 | 20 kDa | 2 |
| predicted protein | 977 | 17 kDa | 2 |
| conserved hypothetical protein | 1773 | 18 kDa | 2 |
| predicted protein | 1375 | 9 kDa | 2 |
| amino acid/cation symporter | 692 | 49 kDa | 2 |
| glutamine amidotransferase | 831 | 25 kDa | 2 |
| ATP-dependent Clp protease adaptor protein ClpS | 1607 | 12 kDa | 2 |
| single-stranded DNA-binding protein | 293 | 10 kDa | 2 |
| gp229 | 301 | 28 kDa | 2 |
| major facilitator superfamily transporter protein | 579 | 41 kDa | 2 |
| conserved hypothetical protein | 725 | 14 kDa | 2 |
| transcriptional regulator | 765 | 30 kDa | 2 |
| predicted protein | 804 | 7 kDa | 2 |
| predicted protein | 808 | 9 kDa | 2 |
| MdaB protein | 867 | 20 kDa | 2 |
| arsenic operon regulator | 921 | 14 kDa | 2 |
| ABC-type cobalamin/Fe3+-siderophores transport system | 1270 | 28 kDa | 2 |
| MreD rod shape-determining protein | 1304 | 19 kDa | 2 |
| major facilitator transporter | 1691 | 46 kDa | 2 |
| predicted protein | 699 | 13 kDa | 2 |
| 2-amino-4-hydroxy-6-hydroxymethyldihydropteridine pyrophosphokinase | 1517 | 18 kDa | 2 |
| predicted protein | 605 | 21 kDa | 2 |
| predicted protein | 1800 | 17 kDa | 2 |
| 5-nucleotidase | 451 | 37 kDa | 3 |
| conserved hypothetical protein | 636 | 21 kDa | 3 |
| predicted protein | 858 | 30 kDa | 3 |
| transcription elongation factor and transcript cleavage | 1338 | 21 kDa | 3 |
| conserved hypothetical protein | 215 | 52 kDa | 3 |
| predicted protein | 27 | 12 kDa | 3 |
| predicted protein | 302 | 9 kDa | 3 |
| IM pore protein | 1958 | 35 kDa | 3 |
| predicted protein | 660 | 21 kDa | 3 |
| outer-membrane lipoprotein carrier protein | 1135 | 23 kDa | 3 |
| trans-aconitate methyltransferase | 519 | 29 kDa | 3 |
| conserved hypothetical protein | 85 | 15 kDa | 2 |
| predicted protein | 87 | 23 kDa | 2 |
| conserved hypothetical protein | 355 | 16 kDa | 3 |
| leucyl/phenylalanyl-tRNA-protein transferase | 1495 | 28 kDa | 2 |
| predicted protein | 562 | 8 kDa | 2 |
| conserved hypothetical protein | 1595 | 22 kDa | 2 |
| oligoketide cyclase/lipid transporter | 28 | 17 kDa | 2 |
| conserved hypothetical protein | 106 | 18 kDa | 2 |
| predicted protein | 308 | 6 kDa | 2 |
| predicted protein | 327 | 13 kDa | 2 |
| ABC-type phosphate transport system | 411 | 31 kDa | 2 |
| conserved hypothetical protein | 495 | 11 kDa | 2 |
| electron transport complex | 509 | 37 kDa | 2 |
| TPR repeat-containing protein | 561 | 34 kDa | 2 |
| conserved hypothetical protein | 686 | 34 kDa | 2 |
| chloride channel protein | 746 | 46 kDa | 2 |
| ser/Thr protein phosphatase | 844 | 41 kDa | 2 |
| predicted protein | 864 | 12 kDa | 2 |
| outer membrane porin protein | 907 | 39 kDa | 2 |
| predicted protein | 1801 | 10 kDa | 2 |
| predicted protein | 1897 | 9 kDa | 2 |
| conserved hypothetical protein | 602 | 10 kDa | 2 |
| conserved hypothetical protein | 883 | 25 kDa | 2 |
| conserved hypothetical protein | 386 | 13 kDa | 2 |
| phosphoribosyltransferase | 1803 | 21 kDa | 2 |
| gp7 | 325 | 12 kDa | 2 |
| predicted protein | 1140 | 9 kDa | 2 |
| 5-nitroimidazole antibiotic resistance protein | 777 | 18 kDa | 2 |
| predicted protein | 635 | 9 kDa | 2 |
| transcriptional regulator | 480 | 8 kDa | 2 |
| predicted protein | 877 | 27 kDa | 2 |
| glycoside hydrolase | 720 | 18 kDa | 2 |
| conserved hypothetical protein | 1861 | 22 kDa | 2 |
| sodium/glutamate symporter | 111 | 51 kDa | 2 |
| conserved hypothetical protein | 252 | 19 kDa | 2 |
| predicted protein | 426 | 23 kDa | 2 |
| predicted protein | 460 | 6 kDa | 2 |
| conserved hypothetical protein | 541 | 39 kDa | 2 |
| predicted protein | 596 | 7 kDa | 2 |
| predicted protein | 624 | 8 kDa | 2 |
| conserved hypothetical protein | 698 | 19 kDa | 2 |
| predicted protein | 799 | 20 kDa | 2 |
| apurinic endonuclease Apn1 | 897 | 30 kDa | 2 |
| spore coat U domain-containing protein | 912 | 33 kDa | 2 |
| conserved hypothetical protein | 984 | 14 kDa | 2 |
| MotA/TolQ/ExbB proton channel family protein | 1048 | 27 kDa | 2 |
| conserved hypothetical protein | 1158 | 32 kDa | 2 |
| GufA protein | 1390 | 33 kDa | 2 |
| membrane protein mma_0477 | 1502 | 22 kDa | 2 |
| glutamate racemase | 289 | 31 kDa | 2 |
| cobalamin biosynthesis protein CobD | 1469 | 36 kDa | 2 |
| conserved hypothetical protein | 333 | 20 kDa | 2 |
| conserved hypothetical protein | 967 | 45 kDa | 2 |
| predicted protein | 144 | 13 kDa | 2 |
| predicted protein | 737 | 7 kDa | 2 |
| predicted protein | 408 | 10 kDa | 2 |
| conserved hypothetical protein | 1946 | 19 kDa | 2 |
| methylated-DNA-protein-cysteine methyltransferase 1 | 1021 | 16 kDa | 2 |
| conserved hypothetical protein | 1003 | 15 kDa | 2 |
| preprotein translocase subunit SecE | 1984 | 13 kDa | 2 |
| chloride channel protein | 1977 | 48 kDa | 2 |
| cellulosome enzyme | 798 | 60 kDa | 2 |
| phosphoribosyl-AMP cyclohydrolase | 1885 | 13 kDa | 2 |
