# Supplementary material for: Proteome Dynamics of the Specialist Oxalate Degrader Oxalobacter formigenes
Source: J Proteomics Bioinform. Author manuscript; Available in PMC 2016 Feb 24. (PMC4764995; doi:10.4172/jpb.1000384)
Supplement: Suppl Table 2 [file NIHMS758057-supplement-Suppl_Table_2.docx]

**Supplementary Table 2**: Relative fold change of proteins that changed significantly between log and stationary phase in *O. formigenes* OxCC13 cultures.

| **Protein Name** | **Accession #** | **Predicted MW** | **Function Prediction** | **Predicted Membrane Association** | **T-test** | **Relative Fold Change (Log versus Stationary)** |
| --- | --- | --- | --- | --- | --- | --- |
| peptidylprolyl isomerase | OFBG_00644 | 18 kDa | Posttranslational modification, protein turnover, chaperones | Yes | 0.04 | -4.58 |
| malonate decarboxylase gamma subunit | OFBG_00828 | 31 kDa | Lipid transport and metabolism | Yes | 0.06 | -3.62 |
| protein-tyrosine-phosphatase | OFBG_00779 | 17 kDa | Signal transduction mechanisms | No | 0.01 | -3.53 |
| outer membrane autotransporter barrel domain-containing protein | OFBG_01404 | 90 kDa | Unknown | Yes | 0.01 | -2.80 |
| NUDIX hydrolase | OFBG_01470 | 26 kDa | Nucleotide transport and metabolism | No | 0.01 | -2.79 |
| maf protein | OFBG_00173 | 22 kDa | Cell cycle control, cell division, chromosome partitioning | No | 0.02 | -2.76 |
| carboxylesterase bioH | OFBG_00951 | 27 kDa | Coenzyme transport and metabolism | No | 0.00 | -2.76 |
| deoxyribodipyrimidine photo-lyase type I | OFBG_00351 | 58 kDa | Replication, recombination and repair | No | 0.02 | -2.75 |
| cell division-specific peptidoglycan biosynthesis regulator FtsW | OFBG_01651 | 45 kDa | Cell cycle control, cell division, chromosome partitioning | Yes | 0.01 | -2.69 |
| cyclic pyranopterin monophosphate synthase subunit MoaC (EC 4.1.99.18) | OFBG_01259 | 18 kDa | Coenzyme transport and metabolism | No | 0.03 | -2.66 |
| nitroreductase | OFBG_00377 | 22 kDa | Energy production and conversion | No | 0.01 | -2.62 |
| resistance protein | OFBG_01759 | 15 kDa | Defense mechanisms | No | 0.01 | -2.57 |
| YceG-like family | OFBG_00943 | 36 kDa | Cell cycle control, cell division, chromosome partitioning | No | 0.02 | -2.54 |
| heavy metal translocating P-type ATPase | OFBG_00530 | 67 kDa | Inorganic ion transport and metabolism | Yes | 0.02 | -2.48 |
| OstA organic solvent tolerance protein | OFBG_01546 | 85 kDa | Defense mechanisms | No | 0.00 | -2.46 |
| Holliday junction DNA helicase subunit RuvA | OFBG_01461 | 21 kDa | Replication, recombination and repair | No | 0.05 | -2.36 |
| alcohol dehydrogenase | OFBG_00838 | 37 kDa | Secondary metabolites biosynthesis, transport and catabolism | No | 0.01 | -2.27 |
| thioesterase | OFBG_01426 | 16 kDa | Lipid transport and metabolism | No | 0.01 | -2.26 |
| transcriptional regulator, TetR family | OFBG_00136 | 24 kDa | Transcription | No | 0.01 | -2.24 |
| TPR repeat-containing protein | OFBG_00553 | 24 kDa | Unknown | Yes | 0.02 | -2.23 |
| DNA-binding response regulator | OFBG_01663 | 26 kDa | Unknown | No | 0.05 | -2.23 |
| nitroreductase | OFBG_01928 | 25 kDa | Energy production and conversion | No | 0.00 | -2.22 |
| glucose-6-phosphate isomerase | OFBG_00140 | 61 kDa | Carbohydrate transport and metabolism | No | 0.02 | -2.21 |
| dephospho-CoA kinase | OFBG_01787 | 23 kDa | Coenzyme transport and metabolism | No | 0.03 | -2.17 |
| PhyA2 protein | OFBG_00852 | 45 kDa | Lipid transport and metabolism | Yes | 0.05 | -2.16 |
| 3-octaprenyl-4hydroxybenzoate decarboxylase (EC 4.1.1.-) | OFBG_01584 | 56 kDa | Coenzyme transport and metabolism | No | 0.01 | -2.16 |
| deoxyguanosinetriphosphate triphosphohydrolase | OFBG_00599 | 51 kDa | Nucleotide transport and metabolism | No | 0.01 | -2.16 |
| ADP-heptose synthase | OFBG_01707 | 17 kDa | Unknown | No | 0.08 | -2.15 |
| thymidylate kinase (EC 2.7.4.9) | OFBG_00942 | 24 kDa | Nucleotide transport and metabolism | No | 0.02 | -2.12 |
| Ku domain-containing protein | OFBG_00801 | 30 kDa | Replication, recombination and repair | No | 0.02 | -2.11 |
| DsrE/DsrF-like family protein | OFBG_00874 | 13 kDa | Inorganic ion transport and metabolism | No | 0.03 | -2.10 |
| transcriptional regulator | OFBG_01550 | 34 kDa | Transcription | No | 0.01 | -2.09 |
| Sel1 repeat-containing protein | OFBG_00585 | 29 kDa | Unknown | No | 0.01 | -2.09 |
| outer-membrane lipoprotein carrier protein | OFBG_01135 | 23 kDa | Cell wall/membrane/envelope biogenesis | No | 0.04 | -2.08 |
| NUDIX hydrolase | OFBG_00841 | 24 kDa | Nucleotide transport and metabolism | No | 0.01 | -2.07 |
| molybdopterin molybdochelatase (EC 2.10.1.1) | OFBG_00896 | 46 kDa | Coenzyme transport and metabolism | Yes | 0.03 | -2.07 |
| pentapeptide repeat-containing protein | OFBG_00446 | 22 kDa | Unknown | No | 0.01 | -2.06 |
| iron-containing alcohol dehydrogenase | OFBG_00792 | 42 kDa | Energy production and conversion | No | 0.01 | -2.06 |
| phosphoglucomutase | OFBG_01422 | 59 kDa | Carbohydrate transport and metabolism | No | 0.04 | -2.06 |
| magnesium and cobalt efflux protein CorC | OFBG_00614 | 33 kDa | Inorganic ion transport and metabolism | No | 0.06 | -2.05 |
| succinyldiaminopimelate desuccinylase (EC 3.5.1.18) | OFBG_01207 | 41 kDa | Amino acid transport and metabolism | No | 0.03 | -2.05 |
| plasmid maintenance system antidote protein | OFBG_00577 | 11 kDa | Defense mechanisms | No | 0.02 | -2.04 |
| aldo/keto reductase | OFBG_00429 | 37 kDa | Energy production and conversion | No | 0.00 | -2.00 |
| ADP-heptose:LPS heptosyltransferase II | OFBG_00221 | 39 kDa | Cell wall/membrane/envelope biogenesis | No | 0.05 | -1.99 |
| outer membrane autotransporter barrel domain-containing protein (copper resistance protein) | OFBG_01218 | 67 kDa | Defense mechanisms | Yes | 0.01 | -1.95 |
| GTPase | OFBG_01299 | 38 kDa | Coenzyme transport and metabolism | No | 0.03 | -1.95 |
| dihydropyrimidine dehydrogenase | OFBG_00487 | 43 kDa | Nucleotide transport and metabolism | No | 0.04 | -1.93 |
| NADH dehydrogenase subunit E (EC 1.6.5.3) | OFBG_01368 | 17 kDa | Energy production and conversion | No | 0.03 | -1.90 |
| phospholipid-binding protein, PBP family | OFBG_00098 | 18 kDa | General function prediction only | No | 0.00 | -1.89 |
| S-adenosyl-methyltransferase MraW | OFBG_01658 | 35 kDa | Cell cycle control, cell division, chromosome partitioning | No | 0.06 | -1.88 |
| cytidylate kinase (EC 2.7.4.14) | OFBG_01625 | 25 kDa | Nucleotide transport and metabolism | No | 0.03 | -1.88 |
| Sel1 repeat-containing protein | OFBG_00504 | 34 kDa | Signal transduction mechanisms | No | 0.02 | -1.87 |
| pyruvate dehydrogenase subunit E1 | OFBG_01612 | 101 kDa | Energy production and conversion | No | 0.02 | -1.87 |
| Malonate decarboxylase delta subunit (MdcD) | OFBG_00827 | 11 kDa | Lipid transport and metabolism | No | 0.03 | -1.87 |
| dihydroorotate oxidase B, catalytic subunit (EC 1.3.3.1) | OFBG_00486 | 45 kDa | Nucleotide transport and metabolism | No | 0.01 | -1.87 |
| Cd(II)/Pb(II)-responsive transcriptional regulator | OFBG_00959 | 19 kDa | Transcription | No | 0.02 | -1.86 |
| polypeptide deformylase | OFBG_00206 | 20 kDa | Amino acid transport and metabolism | No | 0.05 | -1.86 |
| phosphoribosylanthranilate isomerase (EC 5.3.1.24) | OFBG_01346 | 24 kDa | Amino acid transport and metabolism | No | 0.02 | -1.85 |
| single-stranded DNA-binding protein | OFBG_01393 | 18 kDa | Replication, recombination and repair | No | 0.00 | -1.83 |
| superoxide dismutase | OFBG_01322 | 22 kDa | Inorganic ion transport and metabolism | No | 0.01 | -1.82 |
| triosephosphate isomerase (EC 5.3.1.1) | OFBG_01377 | 27 kDa | Carbohydrate transport and metabolism | No | 0.02 | -1.82 |
| phosphatidylinositol phospholipase C | OFBG_00875 | 41 kDa | Signal transduction mechanisms | No | 0.02 | -1.81 |
| p-Nitrobenzoate reductase | OFBG_01011 | 26 kDa | Energy production and conversion | No | 0.04 | -1.81 |
| ABC-type transport system | OFBG_01898 | 24 kDa | Lipid transport and metabolism | Yes | 0.01 | -1.79 |
| nodulation protein L | OFBG_00964 | 20 kDa | General function prediction only | No | 0.03 | -1.78 |
| single-stranded DNA-binding protein | OFBG_00293 | 10 kDa | Replication, recombination and repair | No | 0.02 | -1.77 |
| ABC transporter permease | OFBG_01052 | 67 kDa | Lipid transport and metabolism | Yes | 0.02 | -1.77 |
| nucleotide-binding protein (YajQ-like universal stress protein) | OFBG_01674 | 18 kDa | Unknown | No | 0.00 | -1.77 |
| chorismate synthase (EC 4.2.3.5) | OFBG_01358 | 40 kDa | Amino acid transport and metabolism | No | 0.00 | -1.74 |
| abi family protein | OFBG_00600 | 37 kDa | Defense mechanisms | No | 0.03 | -1.74 |
| carboxylesterase | OFBG_01963 | 24 kDa | Lipid transport and metabolism | No | 0.00 | -1.73 |
| DNA helicase/exodeoxyribonuclease V, beta subunit (EC 3.1.11.5) | OFBG_00812 | 141 kDa | Replication, recombination and repair | No | 0.03 | -1.70 |
| Phosphopantetheine adenylyltransferase (EC 2.7.7.3) | OFBG_02064 | 19 kDa | Coenzyme transport and metabolism | No | 0.00 | -1.70 |
| endoribonuclease L-PSP | OFBG_01232 | 16 kDa | Defense mechanisms | No | 0.02 | -1.69 |
| flavoprotein NADH-dependent oxidoreductase | OFBG_01314 | 40 kDa | Energy production and conversion | No | 0.01 | -1.69 |
| ribosome recycling factor | OFBG_00269 | 21 kDa | Translation, ribosomal structure and biogenesis | No | 0.00 | -1.68 |
| aminotransferase | OFBG_01202 | 44 kDa | Amino acid transport and metabolism | No | 0.02 | -1.68 |
| deoxyuridine 5'-triphosphate nucleotidohydrolase (EC 3.6.1.23) | OFBG_00045 | 16 kDa | Defense mechanisms | No | 0.01 | -1.67 |
| Sel1 repeat-containing protein | OFBG_00592 | 33 kDa | Signal transduction mechanisms | Yes | 0.01 | -1.66 |
| alcohol dehydrogenase GroES domain-containing protein | OFBG_01324 | 36 kDa | Carbohydrate transport and metabolism | No | 0.01 | -1.66 |
| Sel1 repeat-containing protein | OFBG_00745 | 36 kDa | Signal transduction mechanisms | Yes | 0.03 | -1.66 |
| NAD-dependent epimerase/dehydratase | OFBG_01483 | 34 kDa | Carbohydrate transport and metabolism | No | 0.01 | -1.64 |
| pyrimidine regulatory protein PyrR | OFBG_01289 | 19 kDa | Nucleotide transport and metabolism | No | 0.03 | -1.64 |
| periplasmic chaperone for outer membrane proteins SurA | OFBG_01547 | 52 kDa | Posttranslational modification, protein turnover, chaperones | No | 0.01 | -1.64 |
| acetyltransferase (GNAT Domain) | OFBG_00820 | 17 kDa | Unknown | No | 0.02 | -1.63 |
| nicotinate-nucleotide adenylyltransferase (EC 2.7.7.18) | OFBG_00231 | 25 kDa | Coenzyme transport and metabolism | No | 0.03 | -1.63 |
| formyl-CoA transferase | OFBG_02073 | 17 kDa | Unknown | No | 0.02 | -1.63 |
| two-component sensor histidine kinase | OFBG_01453 | 99 kDa | Signal transduction mechanisms | Yes | 0.01 | -1.62 |
| molybdopterin synthase subunit MoaD (EC 2.8.1.12) | OFBG_01125 | 10 kDa | Coenzyme transport and metabolism | No | 0.05 | -1.61 |
| histone acetyltransferase HPA2 (GNAT Domain) | OFBG_01915 | 25 kDa | Unknown | No | 0.05 | -1.61 |
| magnesium and cobalt transporter CorA | OFBG_00438 | 44 kDa | Inorganic ion transport and metabolism | Yes | 0.08 | -1.61 |
| iron-sulfur cluster insertion protein ErpA | OFBG_00038 | 13 kDa | Posttranslational modification, protein turnover, chaperones | Yes | 0.01 | -1.61 |
| RNase G | OFBG_00227 | 55 kDa | Translation, ribosomal structure and biogenesis | No | 0.02 | -1.60 |
| rnfC protein | OFBG_00508 | 46 kDa | Energy production and conversion | No | 0.03 | -1.60 |
| UspA protein (universal stress protein) | OFBG_00781 | 34 kDa | Signal transduction mechanisms | No | 0.04 | -1.60 |
| oligoribonuclease | OFBG_01664 | 23 kDa | RNA processing and modification | No | 0.03 | -1.60 |
| esterase (Beta lactamase activity) | OFBG_01018 | 41 kDa | Defense mechanisms | No | 0.04 | -1.59 |
| formyltetrahydrofolate-dependent phosphoribosylglycinamide formyltransferase (EC 2.1.2.2) | OFBG_01043 | 24 kDa | Nucleotide transport and metabolism | No | 0.02 | -1.57 |
| multiphosphoryl transfer protein 1 | OFBG_01695 | 10 kDa | Carbohydrate transport and metabolism | No | 0.02 | -1.56 |
| D-fructose 1,6-bisphosphatase (EC 3.1.3.11) | OFBG_01239 | 38 kDa | Carbohydrate transport and metabolism | No | 0.01 | -1.55 |
| rare lipoprotein B | OFBG_01972 | 23 kDa | Cell wall/membrane/envelope biogenesis | Yes | 0.02 | -1.55 |
| DNA-binding ATP-dependent protease La | OFBG_00099 | 89 kDa | Posttranslational modification, protein turnover, chaperones | No | 0.00 | -1.55 |
| phosphoenolpyruvate synthase (EC 2.7.9.2) | OFBG_00283 | 89 kDa | Carbohydrate transport and metabolism | No | 0.00 | -1.55 |
| deoxyguanosinetriphosphate triphosphohydrolase | OFBG_02030 | 44 kDa | Nucleotide transport and metabolism | No | 0.04 | -1.53 |
| uroporphyrin-III C/tetrapyrrole methyltransferase | OFBG_01907 | 33 kDa | Translation, ribosomal structure and biogenesis | No | 0.01 | -1.51 |
| radical SAM domain-containing protein | OFBG_01748 | 23 kDa | Unknown | No | 0.04 | -1.51 |
| electron transport complex protein | OFBG_00258 | 25 kDa | Energy production and conversion | No | 0.02 | -1.50 |
| universal stress protein family protein | OFBG_00128 | 30 kDa | Signal transduction mechanisms | No | 0.02 | -1.50 |
| DNA helicase/exodeoxyribonuclease V, gamma subunit (EC 3.1.11.5) | OFBG_00813 | 126 kDa | Replication, recombination and repair | No | 0.04 | -1.50 |
| 30S ribosomal subunit protein S13 | OFBG_02018 | 14 kDa | Translation, ribosomal structure and biogenesis | No | 0.04 | 1.51 |
| large subunit ribosomal protein L24 | OFBG_02007 | 11 kDa | Translation, ribosomal structure and biogenesis | No | 0.01 | 1.53 |
| F0F1-type ATP synthase | OFBG_01823 | 50 kDa | Energy production and conversion | No | 0.02 | 1.54 |
| aminotransferase class-III | OFBG_01412 | 51 kDa | Amino acid transport and metabolism | No | 0.07 | 1.55 |
| small subunit ribosomal protein S8 | OFBG_02010 | 14 kDa | Translation, ribosomal structure and biogenesis | No | 0.04 | 1.55 |
| large subunit ribosomal protein L15 | OFBG_02015 | 15 kDa | Translation, ribosomal structure and biogenesis | No | 0.00 | 1.56 |
| small subunit ribosomal protein S7 | OFBG_01993 | 18 kDa | Translation, ribosomal structure and biogenesis | No | 0.00 | 1.57 |
| glycosyl transferase family 2 protein | OFBG_00222 | 29 kDa | Cell wall/membrane/envelope biogenesis | No | 0.03 | 1.59 |
| TPR repeat-containing protein | OFBG_01920 | 35 kDa | Signal transduction mechanisms | No | 0.04 | 1.59 |
| 30S ribosomal subunit protein S15 | OFBG_00107 | 10 kDa | Translation, ribosomal structure and biogenesis | No | 0.01 | 1.60 |
| ATPase | OFBG_00958 | 80 kDa | Inorganic ion transport and metabolism | Yes | 0.05 | 1.60 |
| large subunit ribosomal protein L7/L12 | OFBG_01989 | 13 kDa | Translation, ribosomal structure and biogenesis | No | 0.00 | 1.60 |
| tex transcriptional accessory protein | OFBG_01867 | 87 kDa | Transcription | No | 0.04 | 1.60 |
| NADH-quinone oxidoreductase subunit G | OFBG_01366 | 85 kDa | Energy production and conversion | No | 0.01 | 1.60 |
| peptidoglycan-associated lipoprotein | OFBG_01279 | 18 kDa | Cell wall/membrane/envelope biogenesis | Yes | 0.03 | 1.60 |
| small subunit ribosomal protein S4 | OFBG_02020 | 24 kDa | Translation, ribosomal structure and biogenesis | No | 0.01 | 1.61 |
| membrane protease FtsH catalytic subunit (EC 3.4.24.-) | OFBG_01163 | 69 kDa | Posttranslational modification, protein turnover, chaperones | Yes | 0.02 | 1.62 |
| ATP synthase F1 subcomplex alpha subunit | OFBG_01825 | 56 kDa | Energy production and conversion | No | 0.00 | 1.63 |
| small subunit ribosomal protein S1 | OFBG_01626 | 63 kDa | Translation, ribosomal structure and biogenesis | No | 0.00 | 1.66 |
| large subunit ribosomal protein L3 | OFBG_01996 | 23 kDa | Translation, ribosomal structure and biogenesis | No | 0.00 | 1.67 |
| 50S ribosomal subunit protein L6/L9E | OFBG_02011 | 19 kDa | Translation, ribosomal structure and biogenesis | No | 0.00 | 1.67 |
| preprotein translocase SecD subunit | OFBG_01850 | 68 kDa | Intracellular trafficking, secretion, and vesicular transport | Yes | 0.03 | 1.67 |
| N6-adenine-specific DNA methylase | OFBG_01969 | 44 kDa | Translation, ribosomal structure and biogenesis | No | 0.03 | 1.68 |
| bifunctional folylpolyglutamate synthase/dihydrofolate synthase | OFBG_01342 | 48 kDa | Coenzyme transport and metabolism | No | 0.12 | 1.70 |
| biopolymer transporter ExbB | OFBG_02038 | 23 kDa | Intracellular trafficking, secretion, and vesicular transport | Yes | 0.04 | 1.71 |
| F0F1 ATP synthase subunit A | OFBG_01829 | 31 kDa | Energy production and conversion | No | 0.01 | 1.72 |
| LSU ribosomal protein L13P | OFBG_00041 | 16 kDa | Translation, ribosomal structure and biogenesis | No | 0.01 | 1.72 |
| large subunit ribosomal protein L4 | OFBG_01997 | 23 kDa | Translation, ribosomal structure and biogenesis | No | 0.00 | 1.73 |
| M50 family peptidase | OFBG_01170 | 25 kDa | Posttranslational modification, protein turnover, chaperones | Yes | 0.04 | 1.73 |
| preprotein translocase YidC subunit | OFBG_01791 | 63 kDa | Cell wall/membrane/envelope biogenesis | Yes | 0.02 | 1.73 |
| small subunit ribosomal protein S21 | OFBG_01320 | 9 kDa | Translation, ribosomal structure and biogenesis | No | 0.02 | 1.74 |
| NADPH-dependent fmn reductase | OFBG_01150 | 21 kDa | Energy production and conversion | No | 0.03 | 1.74 |
| DNA mismatch repair protein mutL | OFBG_01061 | 70 kDa | Replication, recombination and repair | No | 0.03 | 1.77 |
| tRNA pseudouridine synthase B (EC 4.2.1.70) | OFBG_01223 | 35 kDa | Translation, ribosomal structure and biogenesis | No | 0.03 | 1.77 |
| DNA polymerase III subunit gamma | OFBG_01583 | 66 kDa | Replication, recombination and repair | No | 0.01 | 1.79 |
| large subunit ribosomal protein L17 | OFBG_02022 | 15 kDa | Translation, ribosomal structure and biogenesis | No | 0.00 | 1.79 |
| myo-inositol-1 | OFBG_01093 | 29 kDa | Carbohydrate transport and metabolism | No | 0.04 | 1.80 |
| small subunit ribosomal protein S3 | OFBG_02002 | 31 kDa | Translation, ribosomal structure and biogenesis | No | 0.01 | 1.80 |
| SPFH domain, Band 7 family protein | OFBG_00285 | 34 kDa | Energy production and conversion | Yes | 0.06 | 1.80 |
| GTP-binding elongation factor family protein | OFBG_01222 | 68 kDa | Signal transduction mechanisms | No | 0.03 | 1.82 |
| 50S ribosomal protein L29 | OFBG_02004 | 7 kDa | Translation, ribosomal structure and biogenesis | No | 0.01 | 1.82 |
| transcription elongation factor protein | OFBG_01166 | 17 kDa | Transcription | No | 0.02 | 1.83 |
| Sel1 repeat-containing protein | OFBG_00653 | 59 kDa | Signal transduction mechanisms | Yes | 0.04 | 1.84 |
| large subunit ribosomal protein L16 | OFBG_02003 | 16 kDa | Translation, ribosomal structure and biogenesis | No | 0.03 | 1.84 |
| transcriptional regulator | OFBG_00850 | 35 kDa | Transcription | No | 0.00 | 1.85 |
| large subunit ribosomal protein L21 | OFBG_01527 | 12 kDa | Translation, ribosomal structure and biogenesis | No | 0.00 | 1.86 |
| major facilitator transporter | OFBG_00764 | 42 kDa | Inorganic ion transport and metabolism | Yes | 0.13 | 1.87 |
| Na+/solute symporter | OFBG_01951 | 53 kDa | Amino acid transport and metabolism | Yes | 0.12 | 1.88 |
| outer membrane porin protein | OFBG_00365 | 40 kDa | Cell wall/membrane/envelope biogenesis | Yes | 0.05 | 1.89 |
| large subunit ribosomal protein L19 | OFBG_01471 | 15 kDa | Translation, ribosomal structure and biogenesis | No | 0.00 | 1.91 |
| Sel1 repeat-containing protein | OFBG_01374 | 26 kDa | Unknown | No | 0.01 | 1.92 |
| adenine phosphoribosyltransferase (EC 2.4.2.7) | OFBG_01671 | 19 kDa | Nucleotide transport and metabolism | No | 0.03 | 1.93 |
| iron-containing alcohol dehydrogenase | OFBG_00597 | 44 kDa | Energy production and conversion | No | 0.04 | 1.94 |
| replicative DNA helicase | OFBG_01556 | 51 kDa | Replication, recombination and repair | No | 0.00 | 1.95 |
| large subunit ribosomal protein L10 | OFBG_01988 | 18 kDa | Translation, ribosomal structure and biogenesis | No | 0.01 | 1.96 |
| dTDP-6-deoxy-D-xylo-hex-3-ulose aminase | OFBG_01065 | 41 kDa | Cell wall/membrane/envelope biogenesis | No | 0.06 | 1.96 |
| mitochondrial import inner membrane translocase | OFBG_01949 | 33 kDa | Lipid transport and metabolism | Yes | 0.03 | 1.98 |
| DNA helicase | OFBG_00476 | 226 kDa | Replication, recombination and repair | No | 0.03 | 2.00 |
| methylase | OFBG_02063 | 25 kDa | Translation, ribosomal structure and biogenesis | No | 0.01 | 2.05 |
| large subunit ribosomal protein L2 | OFBG_01999 | 30 kDa | Translation, ribosomal structure and biogenesis | No | 0.01 | 2.07 |
| dimethyladenosine transferase dimethyltransferase | OFBG_01549 | 29 kDa | Translation, ribosomal structure and biogenesis | No | 0.05 | 2.08 |
| heat-inducible transcriptional repressor | OFBG_02048 | 37 kDa | Transcription | No | 0.02 | 2.08 |
| Sel1 repeat-containing protein | OFBG_00639 | 44 kDa | Signal transduction mechanisms | Yes | 0.03 | 2.10 |
| N-acetylmuramoyl-L-alanine amidase | OFBG_01508 | 48 kDa | Cell wall/membrane/envelope biogenesis | Yes | 0.01 | 2.12 |
| condensin subunit ScpA | OFBG_01267 | 32 kDa | Replication, recombination and repair | No | 0.03 | 2.12 |
| HAD family hydrolase | OFBG_00514 | 27 kDa | Coenzyme transport and metabolism | No | 0.02 | 2.12 |
| large subunit ribosomal protein L1 | OFBG_01987 | 24 kDa | Translation, ribosomal structure and biogenesis | No | 0.00 | 2.14 |
| exoribonuclease R | OFBG_01569 | 74 kDa | Transcription | No | 0.00 | 2.15 |
| large subunit ribosomal protein L23 | OFBG_01998 | 12 kDa | Translation, ribosomal structure and biogenesis | No | 0.00 | 2.16 |
| DNA helicase II/ATP-dependent DNA helicase | OFBG_01631 | 85 kDa | Replication, recombination and repair | No | 0.01 | 2.20 |
| phosphoribosylaminoimidazole-succinocarboxamide synthase | OFBG_01197 | 33 kDa | Nucleotide transport and metabolism | No | 0.00 | 2.21 |
| GTP-dependent nucleic acid-binding protein EngD | OFBG_02067 | 40 kDa | Translation, ribosomal structure and biogenesis | No | 0.00 | 2.24 |
| ATP-dependent DNA helicase Rep | OFBG_01931 | 77 kDa | Replication, recombination and repair | No | 0.03 | 2.24 |
| outer membrane transport energization protein ExbB (TC 2.C.1.1.1) | OFBG_01048 | 27 kDa | Intracellular trafficking, secretion, and vesicular transport | Yes | 0.08 | 2.25 |
| DNA polymerase III subunit epsilon | OFBG_00982 | 27 kDa | Replication, recombination and repair | No | 0.03 | 2.26 |
| large subunit ribosomal protein L20 | OFBG_00151 | 13 kDa | Translation, ribosomal structure and biogenesis | No | 0.00 | 2.29 |
| sorbitol dehydrogenase | OFBG_01069 | 38 kDa | Secondary metabolites biosynthesis, transport and catabolism | No | 0.05 | 2.31 |
| lipopolysaccharide 3-alpha-galactosyltransferase | OFBG_00226 | 36 kDa | Cell wall/membrane/envelope biogenesis | No | 0.00 | 2.33 |
| ribosomal large subunit pseudouridine synthase B | OFBG_00256 | 56 kDa | Translation, ribosomal structure and biogenesis | No | 0.01 | 2.37 |
| ABC transporter | OFBG_01903 | 62 kDa | General function prediction only | No | 0.00 | 2.42 |
| membrane-fusion protein | OFBG_00591 | 37 kDa | Unknown | Yes | 0.07 | 2.46 |
| UDP-3-O-[3-hydroxymyristoyl] N-acetylglucosamine deacetylase (EC 3.5.1.108) | OFBG_01644 | 35 kDa | Cell wall/membrane/envelope biogenesis | No | 0.03 | 2.49 |
| septum formation initiator | OFBG_00926 | 16 kDa | Cell cycle control, cell division, chromosome partitioning | Yes | 0.04 | 2.55 |
| ribonuclease E protein | OFBG_00889 | 108 kDa | Translation, ribosomal structure and biogenesis | No | 0.00 | 2.57 |
| 2-oxoglutarate dehydrogenase E2 component | OFBG_00454 | 50 kDa | Energy production and conversion | No | 0.00 | 2.57 |
| ATP-binding ABC transporter | OFBG_01525 | 72 kDa | General function prediction only | Yes | 0.01 | 2.68 |
| cation-transporting P-ATPase | OFBG_00749 | 99 kDa | Inorganic ion transport and metabolism | Yes | 0.02 | 2.72 |
| phage tail collar protein | OFBG_00656 | 39 kDa | Unknown | No | 0.05 | 2.75 |
| toxin ABC transporter ATPase and permease | OFBG_00143 | 80 kDa | Intracellular trafficking, secretion, and vesicular transport | Yes | 0.02 | 2.75 |
| ribonucleoside-triphosphate reductase class III catalytic subunit (EC 1.17.4.2) | OFBG_01746 | 78 kDa | Nucleotide transport and metabolism | No | 0.01 | 3.00 |
| transglutaminase domain-containing protein | OFBG_00447 | 24 kDa | Unknown | No | 0.00 | 3.19 |
| FtsZ cell division protein | OFBG_01645 | 42 kDa | Cell cycle control, cell division, chromosome partitioning | No | 0.00 | 3.87 |
| biopolymer transporter | OFBG_02039 | 16 kDa | Intracellular trafficking, secretion, and vesicular transport | Yes | 0.01 | 4.14 |
| membrane-associated metalloprotease | OFBG_00273 | 50 kDa | Unknown | Yes | 0.03 | 4.83 |
| ABC transporter ATP-binding protein | OFBG_01114 | 60 kDa | General function prediction only | Yes | 0.01 | 5.02 |
| AAA-type ATPase | OFBG_00088 | 62 kDa | Unknown | No | 0.00 | 5.44 |
| pyrophosphate-energized proton pump (H(+)-PPase) | OFBG_02044 | 75 kDa | Energy production and conversion | Yes | 0.04 | 7.78 |
